# Multimodal AI for Real‐Time Food Safety and Quality: From Sensors to Foundation Models, Edge Deployment, and Regulation

**DOI:** 10.1002/fsn3.71534

**Published:** 2026-02-19

**Authors:** Zhaojie Chen, Guangyu Zhang, Fan Zhang

**Affiliations:** ^1^ Guangzhou College of Technology and Business Guangzhou People's Republic of China; ^2^ The Affiliated High School of South China Normal University International Department Guangzhou People's Republic of China; ^3^ STS Sugar Company Limited Hong Kong

**Keywords:** biosensing, food quality, food safety, machine learning, near‐infrared, sensors, spectroscopy

## Abstract

Real‐time assurance of food safety and quality requires decisions at line speed, from farm to retail, using signals that span vision, spectroscopy, volatiles, biosensing, and process telemetry. This review investigates and summarizes evidence on multimodal artificial intelligence that fuses such heterogeneous data to detect hazards, verify authenticity, and predict freshness within seconds. We outline sensing coverage along the chain, typical response times, and reported limits of detection, then detail data engineering practices that make disparate streams analysis‐ready, including time synchronization, co‐registration to ground truth, and robust sampling for multisite and multiseason generalization. We appraise fusion strategies, from early and late schemes to attention‐based hybrids that learn joint embeddings across images, spectra, and gas sensor time series, and we summarize head‐to‐head studies where multimodality improves accuracy or reduces error against unimodal baselines. We discuss the maturation of foundation scale encoders and vision language systems for food tasks, together with efficient adaptation, knowledge infusion from HACCP, and bias control. Finally, we examine edge deployment and validation in industrial settings, including hardware constraints, latency budgets, repeatability and reproducibility, documentation for audits, and perspectives on regulatory alignment in EU and US contexts, extended to China's standards‐driven framework where the National Health Commission (NHC) and the State Administration for Market Regulation (SAMR) jointly issue and update National Food Safety Standards (GB) that govern key compliance requirements for labelling and contaminant limits. Evidence gaps persist, notably few multisite deployments over long durations, limited public benchmarks for hyperspectral and e‐nose fusion, and sparse cost–benefit analyses in the scholarly record. Addressing these gaps will enable trustworthy, auditable multimodal AI that complements existing controls and reduces waste while protecting consumers.

## Graphical abstract

The graphical abstract depicts an integrated multimodal AI pipeline for real‐time food safety and quality across the farm‐to‐fork continuum, where heterogeneous sensing modalities including vision, spectroscopy, electronic nose volatiles, biosensing, and IoT/RFID generate complementary data streams that undergo dataset engineering through synchronization, co‐registration, ground truthing, robust multisite and multiseason sampling, and drift monitoring. These processed data are integrated using multimodal AI architectures that combine fusion strategies, foundation models, efficient adaptation, and HACCP‐informed knowledge to enable robust representation learning and decision support. The resulting models are deployed at the edge to deliver low‐latency, uncertainty‐aware, real‐time decisions for hazard detection, authenticity verification, and freshness or shelf‐life prediction, supporting HACCP‐aligned operational actions including rejection, holding, alerting, and recalibration. A continuous feedback loop ensures adaptive updating of data and models, while lifecycle validation and regulatory alignment embed performance evaluation, reproducibility, traceability, human oversight, and compliance with regulatory frameworks in the European Union, United States, and China (GB standards under NHC/SAMR), thereby enabling scalable, trustworthy, and regulation‐ready deployment of multimodal AI in food systems.

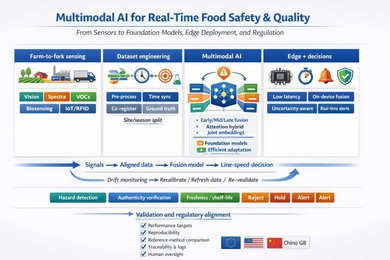

## 1 Introduction

Food safety and food quality are interrelated but distinct concepts. Food quality refers to attributes like nutritional value, freshness, appearance, and taste that meet consumer expectations, whereas food safety focuses on the absence of biological, chemical, or physical hazards that could harm consumers (Nikzadfar et al. 2024). In practice, large batches of substandard product may be produced before quality problems are noticed, and undetected safety hazards can lead to foodborne illness (Aviara et al. 2022; Guo et al. 2023). Ensuring both safety and quality in real time is therefore critical. Hereby, “real‐time” implies monitoring and decisions at industrial line speeds (often within seconds) such that contaminated or noncompliant items can be identified and removed without slowing production.

Traditionally, food quality and safety inspection relied on manual sampling and lab tests or human visual inspection; these methods, while trusted, are laborious, slow (minutes to days for lab assays), and prone to human error or fatigue (Pan 2025). Over the past few decades, the field has progressed through a *historical arc* from manual inspection, to early machine vision and single‐sensor automation in the 1990s–2000s, to the adoption of deep learning in the 2010s, which dramatically improved the accuracy of image‐based and sensor‐based detections (Chen et al. 2024; Chen and Yu 2022; Ekramirad et al. 2024; Li et al. 2023; Siripatrawan and Makino 2024; Zhao et al. 2023). For example, classical computer vision methods for fruit inspection could detect surface defects, but modern deep learning can also infer internal rot or disease from subtle external cues with much higher precision (Nikzadfar et al. 2024). In parallel, recent advances in nanomaterial‐enabled electrochemical sensing have improved rapid, on‐site detection of additives and residues in complex matrices, complementing optical and spectroscopic modalities in practical monitoring workflows (Murugesan et al. 2025; Sakthivel et al. 2024; Stanley et al. 2025).

Despite these advances, single‐modality systems have inherent blind spots: *no one sensor sees everything*. This has motivated the recent rise of multimodal AI systems that fuse data from multiple sensor types to provide a more holistic and robust assessment (Min et al. 2025; Xiao et al. 2025). Multimodal approaches leverage the fact that different sensing principles have complementary strengths and failure modes. For instance, optical imaging excels at catching visible defects or foreign objects, while spectroscopy can detect molecular composition changes invisible to cameras; electronic noses sniff spoilage volatiles that optical or spectral sensors might miss, and so on (Xiao et al. 2025). By combining modalities, errors can be reduced; what one sensor misses might be caught by another. Recent systematic reviews confirm that multimodal sensor fusion often outperforms single‐sensor methods for food quality and safety monitoring (Fendor et al. 2025; Zhang et al. 2025).

However, designing effective multimodal food monitoring systems presents significant challenges. Different data types like images, spectra, chemical sensor readings, etc., have disparate formats and sampling rates, complicating data alignment and fusion (Bannach et al. 2009; Multimodal Data Fusion—an Overview|ScienceDirect Topics, n.d.). Models may suffer covariate shift when deployed: for example, a model trained on one season's harvest or a specific processing plant may falter under different crop conditions or in a new facility (Dias Cappelini et al. 2025; Wedding et al. 2013). Domain drift is also common: Sensors themselves can drift (such as an odor sensor's baseline shifts over months) and food profiles evolve (like new variants, suppliers, or recipes) (Wörner et al. 2025). Ensuring generalization across sites and seasons requires large, representative datasets and often adaptation strategies (Dias Cappelini et al. 2025; Wedding et al. 2013). Moreover, data heterogeneity and volume pose engineering hurdles: synchronizing high‐speed vision data with slower chemical sensor data and filtering noise demand robust data pipelines. In practice, the food industry also faces constraints around privacy and data sharing, since production data may be proprietary; an issue that multimodal AI systems must navigate via federated learning solutions (Gavai et al. 2023). Despite these challenges, the potential benefits, a proactive, automated “eye‐nose” on the process that can catch problems in real time, drive intense research and innovation in this area.

**FIGURE 1:**
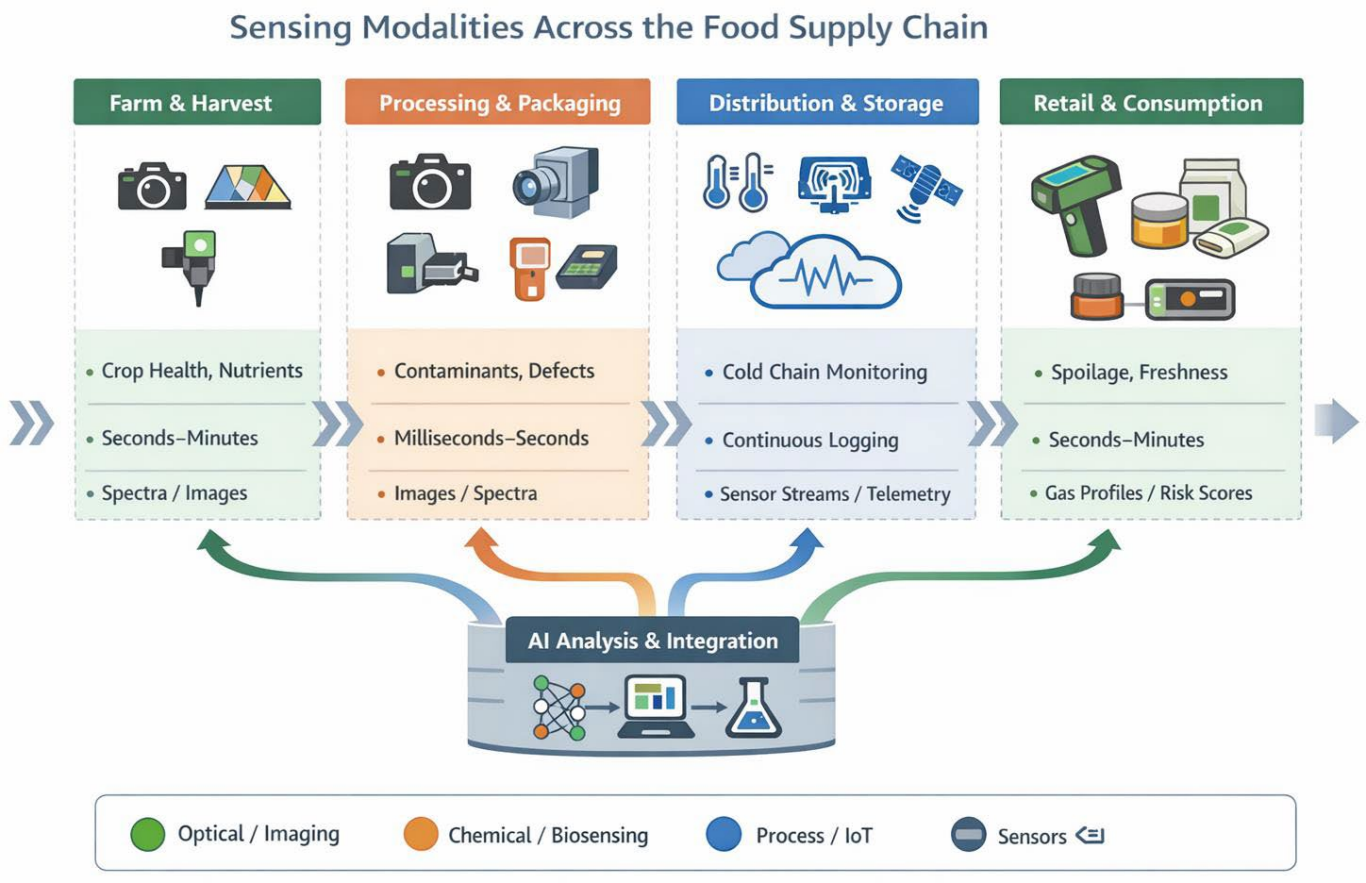
Typical deployment of sensing modalities across the food supply chain. Each stage applies different sensor types to capture relevant safety and quality attributes. On farms, optical and spectroscopic tools assess crop health, composition, and contamination risks. In processing and packaging, high‐speed imaging (RGB, X‐ray, HSI), spectroscopic, electronic‐nose, and biosensing systems detect defects, adulterants, and residues in real time. During distribution and storage, IoT and RFID sensors continuously log temperature, humidity, and gas composition to model cold‐chain integrity and predict spoilage risk. At retail and consumption stages, portable spectroscopy, e‐noses, and smart‐packaging biosensors provide freshness or authenticity assessments. Together, these data streams enable multimodal AI models that integrate visual, chemical, and environmental information for comprehensive food‐safety monitoring.

The goal of this review was to survey the state of the art in multimodal sensing and AI for real‐time food safety and quality assurance. In the following sections, we first discuss the range of sensing modalities available across the farm‐to‐fork chain, then address how the data from these heterogeneous sources are engineered and curated for AI model development. We will also cover multimodal model architectures, deployment at the edge, and the regulatory outlook, respectively. By framing the context, we set the stage for understanding how sensors and data come together as a foundation for intelligent food quality/safety systems (Figure 1).

## 2 Sensing and Data Modalities Across the Food Chain

Modern food quality/safety monitoring employs a spectrum of sensing modalities, each targeting specific attributes. Table 1 provides a comparative overview. In this section, we review five major categories, including imaging, spectroscopic, volatile/gas sensors, biosensors, and process/logistics sensors, outlining what they measure, typical performance, and deployment points along the chain. Emphasis is given to real‐time capabilities such as sensor response times and achievable accuracy or detection limits, as reported in studies (Table 1).

**TABLE 1:** Comparison of sensing modalities for real‐time food safety and quality monitoring.

Modality	Principle	Primary targets	Response time	Performance (typical)	Sample prep	Portability	Cost (relative)^a^	Strengths	Limitations
Visual imaging (RGB, MS, HSI, X‐ray)	Optical features; X‐ray density	Defects, foreign bodies, size/color; internal (X‐ray/HSI)	RGB: ≤ 0.1 s/item; HSI: 1–2 s/item	High (task‐dependent)	None	High	RGB 2×; MS 3×; HSI 4×; X‐ray 5×	Line‐speed, full coverage; interpretable	Surface bias (except X‐ray/HSI); lighting/occlusion; bulky (HSI/X‐ray); high data (HSI)
Spectroscopy (NIR, FTIR, Raman)	Molecular spectra	Composition, adulteration, authenticity; some hazards	Seconds/scan	High (matrix‐dependent)	Low	Medium	Handheld NIR 3×; FTIR 4×; Raman 4×	Rapid chemical readout; often nondestructive	Calibration/transfer; drift; point sampling (unless imaging)
Electronic nose (VOC arrays)	VOC pattern response	Spoilage, freshness, off‐odors	Seconds–minutes	Often high for classification	None (headspace)	High	3×	Fast VOC screening; low‐power options	Drift; humidity/temperature effects; limited specificity
Biosensors (immuno/aptamer/enzyme; LoC, PCR‐on‐chip)	Biorecognition signal	Pathogens, toxins, allergens, residues	Minutes–hours	Very high (assay‐dependent)	Variable	High	Per‐test 1×; reader 2×; LoC or portable PCR 4×	High specificity; detects nonvisible hazards	Consumables; matrix fouling; often single‐analyte; confirmation may be required
Process/IoT sensors (T/H, vibration, RFID)	Environment/process logs	Cold chain, process deviation, logistics, traceability	Continuous	Indirect (model‐based)	N/A	High	Tags 1×; gateway 2×; platform 3×	Preventive plus traceability	No direct product readout; integration and interoperability burden

Abbreviations: FTIR, Fourier‐transform infrared; HSI, hyperspectral imaging; IoT, Internet of Things; LoC, lab‐on‐chip; MS, multispectral; NIR, near‐infrared; PCR, polymerase chain reaction; RFID, radio‐frequency identification; RGB, red–green–blue; T/H, temperature and humidity; VOC, volatile organic compound.

^a^ Cost key: 1× very low cost (often consumables or simple tags); 2× low‐cost device; 3× mid‐cost instrument or platform; 4× high‐cost system; 5× very high‐cost industrial system.

### 2.1 Optical and Imaging Sensor

Optical imaging covers regular Red‐Green‐Blue (RGB) cameras, multispectral/hyperspectral imagers, laser‐based scanners (including X‐ray imaging), fluorescence imaging, and thermal cameras. These sensors detect *visible or spatial defects* and are widely used on processing lines for automated vision inspection (Jin et al. 2023; Pu et al. 2023). Common applications include detecting foreign objects, surface damage or bruising, color and shape sorting, including ripeness or size grading, and indirect signs of contamination, such as fungal growth or fecal contamination spots (Kurniawan et al. 2024). Optical systems are prized for being fast and noncontact. High‐speed line‐scan cameras can inspect tens of items per second; for example, a recent system using the YOLOv5 deep learning model identified foreign fragments on fresh‐cut vegetables with *98.3% accuracy* and as little as 2.6 milliseconds inference time per image (Kurniawan et al. 2024). This illustrates that modern vision systems can keep up with rapid production throughput while maintaining high accuracy.

Hyperspectral imaging (HSI), which captures hundreds of wavelength bands per pixel, adds a chemical dimension to optical inspection. HSI can nondestructively map compositions like moisture or fat and detect defects invisible in RGB, such as early rot or mycotoxin infection inside grains (Nikzadfar et al. 2024). For instance, an HSI system discriminating fungal‐infected cereal kernels achieved > 90% accuracy in classifying infection and > 80% accuracy in classifying specific mycotoxin contamination levels (Femenias et al. 2022). Fluorescence imaging is another optical tool: Many bacteria, molds, and residues fluoresce under UV light, so cameras with appropriate excitation can reveal microbial contamination or bruises via fluorescence signatures in real time (Nikzadfar et al. 2024). Thermal cameras, while less common for quality grading, are used in monitoring cooking or cooling processes, such as ensuring uniform baking or checking for overheated spots.

#### 2.1.1 Deployment, Strengths, and Limitations

Optical sensors are typically mounted on production lines like above conveyor belts for 100% inline inspection of products or used in grading facilities (for produce) and abattoirs (for carcass inspection). They provide immediate pass/fail or sorting decisions. Foreign‐object X‐ray/laser systems can detect contaminants like bone fragments in meat with > 95% accuracy (Andriiashen et al. 2023). Vision‐based fruit sorting can classify fruits by grade with 90%–95% accuracy in many cases (Sanislav et al. 2025). The strength of imaging is high speed and intuitive results (images), but limitations include being mostly superficial (limited insight into internal composition unless using penetrating radiation like X‐ray) and susceptibility to occlusion (e.g., mud covering a defect). Multispectral and HSI devices also tend to be costlier and bulkier than plain RGB cameras, though prices continue to fall.

### 2.2 Spectroscopic Sensors

Spectroscopic methods such as Near‐Infrared (NIR), Mid‐IR (FTIR), and Raman spectroscopy provide a molecular fingerprint of foods (Abdullahi et al. 2026). They detect specific chemical bonds and compositions, making them powerful for assessing nutrient content (e.g., sugar, protein, water), detecting adulterants or contaminants, and verifying authenticity (varietal or geographic origin). Unlike imaging, spectroscopy does not form a spatial picture but rather gives an average spectrum of a sample or even a point measurement (unless combined with imaging in hyperspectral systems).

A classic use case is rapid compositional analysis: For example, portable NIR instruments can predict properties like fat or moisture in meat and dairy within seconds, often with ±0.5% error after calibration (Fodor et al. 2024; Folli et al. 2022). For adulteration detection, NIR or Raman can be highly sensitive. One study using NIR spectroscopy detected melamine adulteration in milk down to very low levels, achieving *100% classification accuracy* for adulterated vs. pure milk by applying a partial least squares discriminant analysis (PLS‐DA) model (Wu et al. 2016).

Generally, chemometric methods (PLS, principal components, etc.) have been the workhorse for quantitative spectral analysis, and they set a strong baseline performance that newer machine learning models aim to surpass. Raman spectroscopy, especially when enhanced with substrates (SERS), can even detect trace contaminants like pesticides or illegal dyes at the ppm to ppb level. For instance, a SERS sensor with nanostructured substrates identified pesticide residues with detection limits in the tens of parts‐per‐billion range (Chi et al. 2020); one report quantified an organochlorine pesticide at 87 ppb using a nanoporous Ag sensor, and another achieved sub‐1 ppb (0.258 ppb for thiram on fruit surfaces) by combining SERS with optimized extraction (Pan, Chen, et al. 2024). These examples underscore the extreme sensitivity possible: by amplifying weak Raman signals, SERS‐based methods can approach regulatory residue limits.

Pre‐processing is critical in spectroscopy; methods like standard normal variate (SNV) or multiplicative scatter correction are routinely applied to remove noise and baseline drift (Nikzadfar et al. 2024; Pan, Chen, et al. 2024). Such steps, along with chemometric modeling (e.g., constructing a PLS calibration curve), are often needed to make spectral data analysis‐ready—effectively, traditional chemometrics form an initial AI that newer deep learning models must outperform.

#### 2.2.1 Deployment, Strengths, and Limitations

Spectrometers are used both in labs (benchtop units for confirmatory analysis) and in‐line or at‐line (fiber‐optic probes, handheld NIR guns, or micro‐FTIR devices for on‐site checks). Response is near‐instantaneous (typically a few seconds per scan). Direct insight into chemical composition and typically no sample prep (for NIR/Raman). It requires calibration for each product matrix; performance can degrade if the instrument or environment changes (necessitating calibration transfer or model updates), and spectra can be influenced by sample heterogeneity (e.g., grain particle size) (Kramer 2005; Workman 2025). Nonetheless, spectroscopy provides an invaluable layer of information that complements imaging—catching invisible adulterants or predicting quality indices like sweetness (via soluble solids content), which imaging alone cannot measure.

### 2.3 Volatile/Gas‐Phase Sensors (Electronic Noses)

Many spoilage and contamination processes emit characteristic volatile compounds. Electronic noses (e‐noses) are sensor arrays that attempt to mimic the human sense of smell by detecting volatile organic compounds (VOCs) released from food (Guo et al. 2020; Sanislav et al. 2025). Typical e‐noses employ a set of nonspecific gas sensors, such as metal‐oxide semiconductor sensors, MOS, or conductive polymer sensors, each with partial sensitivity to different volatiles; the collective response pattern is analyzed via machine learning to identify odors or estimate spoilage levels. E‐noses have shown particular promise for monitoring the freshness/spoilage of meats, fish, fruits, and detecting off‐odors or contaminants like amines from fish spoilage, or fungal aroma from moldy grains (Guo et al. 2020; Madhubhashini et al. 2024). They are also used for authenticity testing, including distinguishing varietal aromas in beverages, and even in packaging leak detection.

A major advantage is speed—e‐noses typically sniff and return results in under a minute. One study combined an e‐nose with a machine learning classifier to predict stored meat freshness and achieved over 90% accuracy in distinguishing fresh vs. spoiled samples (Feng et al. 2020). Another report notes that portable e‐noses have reached accuracies *exceeding 90%* in most cases for quality evaluations in controlled tests (Sanislav et al. 2025). In fact, an AI‐powered e‐nose developed for meats demonstrated about 98%–99% accuracy in classifying freshness grades across beef, chicken, and fish when tested on packaged samples, highlighting that, under laboratory conditions, smell can be a very specific indicator of spoilage progression (Guo et al. 2020).

Despite these successes, sensor drift and calibration remain notorious challenges for e‐noses (Bosch et al. 2022). Metal‐oxide sensors' baselines change over time (months or even days of continuous use), and sensor responses can be affected by humidity, temperature, or sensor aging. Without compensation, an e‐nose model's accuracy can degrade significantly after prolonged use or when applied in a new environment (Liu et al. 2018). Researchers are actively developing drift compensation algorithms—for example, semi‐supervised domain adaptation approaches have been proposed to recalibrate e‐nose data streams periodically. Another issue is selectivity: unlike a dedicated chemical analyzer, e‐noses produce composite signals (like smells) that may be confounded by mixtures. This means extensive training on known aromas is needed, and there is a risk of interference if unexpected volatiles are present.

Nonetheless, modern e‐noses combined with pattern recognition have achieved impressive robustness in many applications. They are often used at *receiving or storage* stages—e.g., sniffing the headspace of milk silos, meat chill rooms, or produce storage to give an early warning of spoilage before human senses would detect it. They also appear in packaging lines (sniffing sealed packages for integrity) and even in the field (sniffing crops for disease markers). Response times are usually on the order of seconds to a minute due to the need for volatile diffusion and sensor equilibration. Many e‐nose devices include internal reference purges and require periodic zeroing with clean air.

E‐noses add a vital sensory modality (“smell”) that catches what eyes and spectrometers might miss. Their high sensitivity to trace gases can even outperform microbial plate counts in early spoilage detection (since volatiles build up before microbial counts are dangerously high). The tradeoff is that maintaining calibration and consistency is nontrivial—in industrial practice, e‐noses may need frequent recalibration or replacement to ensure reliability (Rudnitskaya 2018; Wu et al. 2023). Research continues into more stable sensors and machine learning methods to mitigate drift.

### 2.4 Biosensors and Microfluidic Assays

Biosensors are analytical devices that incorporate a biological recognition element, such as an antibody, enzyme, aptamer, or DNA probe, to selectively detect specific targets as pathogens, allergens, or chemical residues. They often output an electrical or optical signal when the target binds. In food safety, biosensors aim to provide rapid, on‐site alternatives to traditional laboratory tests (like culture plating or ELISA), which can take days. Modern biosensors, including lateral flow immunoassays, enzyme‐based test strips, and lab‐on‐chip microfluidics, can dramatically shorten detection times for pathogens and toxins (Quintela et al. 2022). For instance, lateral flow devices (the same basic format as a home pregnancy test) have been developed for pathogens like *Salmonella*—some can deliver a result in 15 min with a detection limit on the order of 10^4^ colony‐forming units (CFU) per mL (Taitt et al. 2004). By extending assay time to 1 h, the sensitivity in that study improved tenfold to about 10^3^ CFU/mL (Taitt et al. 2004). These timeframes represent a huge improvement over classical culture, which requires 1–3 days of incubation (Samota et al. 2022). Likewise, microfluidic PCR (“lab‐on‐a‐chip”) devices have been shown to detect pathogens in under an hour, including sample preparation. Many biosensors also target allergens and residues: for example, immunosensors for allergens like peanut or gluten can routinely detect contamination at the low ppm (mg/kg) level within minutes (Aquino and Conte‐Junior 2020; Röder et al. 2009). One nanoparticle‐based electrochemical sensor reported detecting peanut allergen down to 0.2 mg/kg (0.2 ppm) in food extracts—well below typical regulatory thresholds—demonstrating how sensitivity continues to improve with nanomaterials and signal amplification (Aquino and Conte‐Junior 2020). Recent electrochemical sensor platforms also report sensitive detection of food additives and contaminants such as vanillin and roxarsone using nanostructured electrodes, supporting faster screening in field and at‐line settings (Gokulkumar et al. 2024; Murugesan et al. 2025). At the extreme, researchers have even achieved *single‐bacterium detection*: an advanced nanomaterial biosensor was able to detect as low as 1 *E. coli* cell in a sample (effectively an ultra‐low limit of detection). Such feats, while demonstrated in lab settings, underscore the potential of biosensors to rival the sensitivity of laboratory methods.

Sample‐to‐answer time is a key metric. Many biosensors integrate sample prep like filtering or preconcentrating the sample, to speed up analysis. “Direct” biosensors (like some aptamer‐based assays) can sometimes be applied to a food swab or liquid without enrichment, yielding results in < 30 min, though often at the cost of a higher detection limit. In contrast, if a few hours can be afforded, incorporating a brief culture enrichment or DNA amplification step can dramatically lower detection limits (catching just a few CFU) (Vikesland and Wigginton 2010). Depending on the need (e.g., a production line CCP might prefer speed over absolute sensitivity), biosensors can be configured accordingly.

#### 2.4.1 Deployment, Strengths, and Limitations

Biosensors and microfluidic kits are frequently used in at‐line or in‐field testing; for example, a quality control technician might take a swab or drip sample from the line and apply it to a test cartridge, getting a result by the time the batch is done. Some biosensors are also integrated into processing equipment, such as real‐time monitoring of cleaning solutions for pathogen load using fluorescence sensors (Heo and Hua 2009). Unparalleled specificity (antibody or DNA probes target a unique pathogen or toxin) and increasingly user‐friendly portable formats. Many require consumables (test strips, reagents) and have a finite shelf‐life; they often address one analyte at a time (unlike e‐noses, which sense broadly), and matrix effects (like food particles clogging a microfluidic channel) can interfere, so sample preparation is sometimes needed. Nonetheless, the trend is toward faster and more multiplexed biosensors—for example, there are chip‐based assays now that can simultaneously screen for multiple pathogens in one go (Heo and Hua 2009). In regulatory contexts, biosensor results often still need confirmation by standard methods, but their real‐time alerts allow processors to act faster (withhold a batch, initiate sanitation) pending confirmation.

### 2.5 Process and Logistics Telemetry

Beyond direct product sensors, a wealth of “contextual” data is collected throughout food production and distribution that indirectly relates to safety and quality. This includes process telemetry (machine settings, line speeds, pressures, etc.), environmental sensors (temperature, humidity, vibration, gas levels in storage), and digital traceability data (timestamps, locations via RFID or barcodes, supply chain records). While these are not food sensors per se, they are essential to a *multimodal approach*—when combined with product observations, they enable predictive analytics and traceability. For example, continuous temperature monitoring in the cold chain is crucial for predicting shelf life: If a perishable experiences temperature abuse, algorithms can calculate the reduction in remaining shelf life (Göransson et al. 2018; Potyrailo et al. 2012).

Studies have shown that using dynamic temperature data to adjust shelf‐life predictions (instead of assuming ideal conditions) significantly improves the accuracy of expiration dating (Gaukler et al. 2017; Wu, Zou, et al. 2025). One field trial with RFID‐based temperature loggers on meat shipments demonstrated that real‐time temperature data enabled *dynamic shelf‐life estimates* that were far more precise than static use‐by dates, allowing producers to extend or shorten shelf life on the fly and potentially reducing waste (Göransson et al. 2018; Zou et al. 2025). Similarly, humidity and vibration sensors during transport can indicate produce stress—excessive vibration might bruise fruits, or low humidity might cause wilting. If telemetry flags a deviation, cloud‐based analytics can predict increased spoilage risk or trigger an alarm to check product quality at receiving. Modern supply chains also use GPS and RFID to track shipments; when a contamination incident occurs, these data allow rapid tracing of affected lots. In manufacturing, line sensors and PLC data (e.g., fill weights, thermal process parameters) can be harnessed by AI models to predict outcomes—for instance, combining cooker temperature profiles with final product microbial tests to build a model that *predicts contamination risk* based on cooker performance anomalies. Administrative data like cleaning schedules, operator logs, and ingredient sources can further enrich this picture.

A key role of these telemetry and traceability data streams is to serve as predictors in multimodal AI models. Unlike sensors that directly measure a hazard, these data reflect conditions that correlate with hazards. For example, a history of mild temperature abuse during transit might not show immediate spoilage markers, but a machine learning model can incorporate that history to predict a higher probability of spoilage onset 2 days sooner than normal (Onyeaka et al. 2025). Likewise, integrating processing parameters (e.g., pasteurization time) with product sensor data can improve contaminant detection by accounting for expected reduction from the process. IoT in Food Logistics is a burgeoning area: battery‐free RFID sensor tags that log temperature and even gas composition in each pallet or container are now available (Hy‐Vee Grocery Cold Chain Monitoring Increases Product Shelf‐Life, n.d.; Potyrailo et al. 2012). These generate big data that, when mined, reveal patterns (e.g., routes or vendors associated with more excursions) and can feed into blockchain or cloud platforms for end‐to‐end visibility. For real‐time control, simple rules like “if any logger exceeds 10°C for > 30 min, alert QA” are used, but more sophisticated approaches use these data in combination—for instance, feeding temperature profiles into predictive microbiology models to estimate microbial growth in situ (Tarlak 2023).

In practice, process and telemetry data are already widely collected (for compliance and process control); the challenge is to integrate them with sensor outputs in a meaningful way. Many food companies are developing digital twins of their supply chain—virtual models that continuously update with sensor and process data—to run simulations and risk predictions (Göransson et al. 2018; Zou et al. 2025).

#### 2.5.1 Deployment, Strengths, and Limitations

These sensors and data systems operate continuously in the background. Temperature and humidity sensors might be embedded in storage rooms, trucks, or even packaging. RFID tags accompany products through the chain, automatically logging data. Production machinery often has built‐in sensors whose data can be tapped via industrial protocols. Humans are rarely directly involved except when an alarm or dashboard indicates a problem. Provides preventive insight (e.g., predicting a problem before it manifests in the product) and enhances traceability for recalls. By themselves, these data don't confirm a hazard—they indicate risk. Also, interoperability and data overload issues arise: aggregating readings from thousands of tags and sensors requires robust IoT infrastructure and analytics. Nevertheless, when combined with direct measurements, process telemetry forms the “glue” of a multimodal food monitoring system, contextualizing sensor findings with the when/where/how of the product's history (Göransson et al. 2018; Potyrailo et al. 2012).

## 3 Data Engineering and Curation for Multimodal Pipelines

**FIGURE 2:**
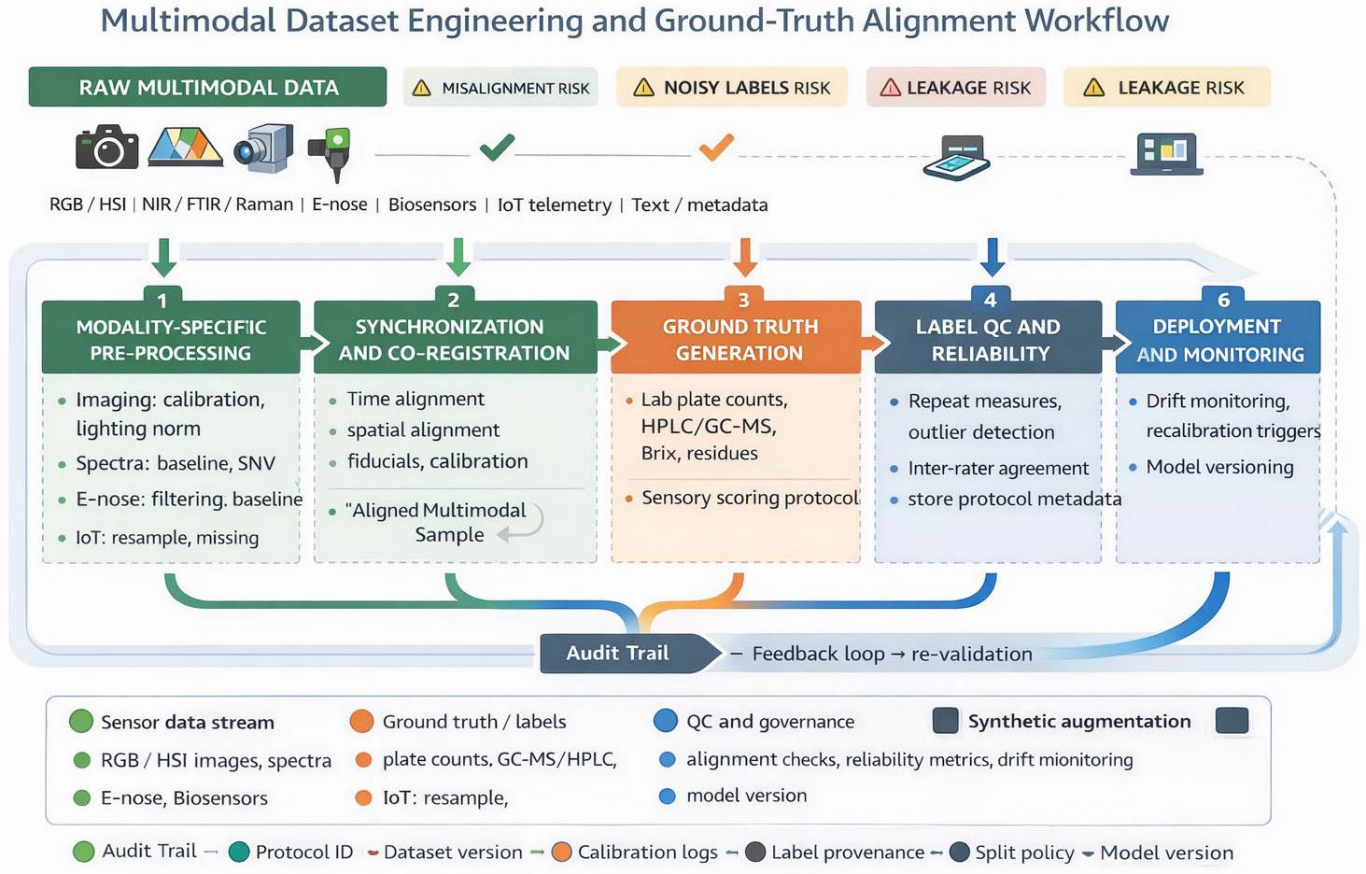
Multimodal dataset engineering and ground‐truth alignment workflow. Workflow for constructing multimodal datasets for food safety and quality AI. Raw asynchronous sensor streams are pre‐processed per modality, then synchronized and co‐registered to create aligned multimodal samples. Ground truth is generated using laboratory reference methods and standardized sensory protocols, followed by label quality control, reliability checks, and retention of provenance metadata. Where synthetic augmentation is used, sim‐to‐real discrepancy verification and task‐grounded utility checks are applied before inclusion. Dataset splits prioritize site, season, and batch separation to reduce leakage, and post‐deployment monitoring closes the loop by detecting drift and triggering recalibration, data refresh, and re‐validation.

Collecting data from multiple sensors is only the first step; transforming these raw streams into *analysis‐ready datasets* is crucial for building robust multimodal AI models (Figure 2). This section covers how heterogeneous data are synchronized and standardized, how ground truth labels are obtained and aligned across modalities, dataset design considerations to ensure models generalize, strategies to handle distribution shift and sensor drift, and data governance issues like privacy and sharing. Proper data engineering underpins the success of multimodal learning—without it, even the most sophisticated model will falter due to garbage‐in/garbage‐out (Table 2).

**TABLE 2:** Notable multimodal food datasets and studies (for safety and quality applications).

Dataset/study (year)	Modalities	Domain	Size	Ground truth (labels)	Tasks	Reported performance	Limitations and notes
Nikzadfar et al. (2024)	HSI + e‐nose (MOS)	Filled pancakes	*n* = 1200 (multibatch)	Authentic vs. adulterated (visual inspection of filling; specific leafy‐green adulterant)	Binary classification	Fusion accuracy > 98% (HSI 95%; e‐nose 92%)	Single food item and one known adulterant; does not test broad adulteration. Public dataset reported.
Lun et al. (2025)	Fluorescence HSI + e‐nose + reference assays	Chilled pork	*n* = 150 (0 to 14 days storage)	TVB‐N (mg/100 g), microbial counts (log CFU), sensory freshness grade	Regression (spoilage); three‐class grading	TVB‐N: R2 0.94, RMSE 2.1 mg/100 g; grade accuracy 96%	Controlled lab storage; drift‐prone sensors (calibration applied); not validated in industrial conditions.
Kabir et al. (2025)	RGB (high‐speed) + depth	Fresh‐cut vegetables	5000 images	Presence and type of foreign material (wood, plastic, metal, etc.), hand‐labeled	Detection (localize + classify)	98.3% detection accuracy; 2.6 ms per image inference	Visible contaminants only; may not generalize to occluded objects; depth helped separate leaves from solid debris.
Fusion of Electronic Nose and Hyperspectral Imaging for Mutton Freshness Detection Using Input‐Modified Convolution Neural Network—SUNY Empire State College, n.d.; Wu, Zhang, et al. (2025)	e‐nose + HSI + structured illumination	Mutton slices	*n* = 240 (4 freshness groups)	TVB‐N, total bacterial count, storage time (0 to 9 days)	TVB‐N regression; freshness‐day classification	TVB‐N: R2 0.91 (e‐nose only 0.85); 0‐day vs. 9‐day classification 100%	Complex lab setup; not directly in‐line; needs validation on real abattoir samples.
Chen et al. (2023)	RFID temperature + humidity + product images	Fruits (kiwi, peach)	*n* = 300 (3 seasons)	Time‐to‐spoilage (expert inspection) + weight loss percentage	Shelf‐life prediction	Remaining shelf‐life within plus or minus 1.5 days (vs plus or minus 4 days with static assumption)	Multi‐season design improves generality; depends on consistent expert spoilage definition.
Gavai et al. (2023)	Distributed spectral + chemical assays + supply‐chain records (no raw sharing)	Olive oil	Total *n* = 800 (across 3 companies)	Adulteration percentage + fraud label (confirmed by lab, e.g., GC–MS profile)	Regression (adulteration); classification (fraud)	Fraud AUC 0.98 (single‐company 0.85); adulteration MAE 1.5%	Requires aligned labeling and compatible feature spaces across partners; demonstrates privacy‐preserving collaboration.

Abbreviations: AUC, area under the curve; CFU, colony‐forming units; HSI, hyperspectral imaging; MAE, mean absolute error; MOS, metal‐oxide semiconductor; RFID, radio‐frequency identification; RMSE, root mean square error; TVB‐N, total volatile basic nitrogen.

### 3.1 Harmonizing Heterogeneous Data Streams

In a multimodal setup, one may have camera images (2D pixel grids), spectral readings (1D wavelength plots), time‐series from e‐noses, and scalar process parameters all describing the *same sample or batch*. Time synchronization and alignment of data is a fundamental task: all data modalities must be referenced to a common timeline or event (e.g., the product ID or timestamp as it passes a certain point). For high‐throughput applications, this can be challenging—a camera might snap an image every 100 milliseconds, while an e‐nose might require a 10‐s sampling cycle. Solutions include triggering sensors simultaneously or interpolating slower sensor data between discrete events (Gaw et al. 2022; Ren et al. 2025). In practice, industrial systems often employ a PLC (programmable logic controller) to send a trigger signal to all sensors when a product is at the inspection station, ensuring data correspondence. When sensors operate at different rates, buffering and time‐stamping each data point allows software to later join the streams by nearest timestamps or by product IDs. For example, in an automated fruit sorter with both an RGB camera and an NIR spectrometer, each fruit can be assigned a unique ID as it enters; the camera image and spectrometer reading are tagged with that ID so they can be merged in the dataset (Soncini et al. 2025).

Beyond timing, spatial registration may be needed. If an RGB camera and an HSI camera view the same object, their images must be spatially aligned (often achieved by calibration using a checkerboard or having one sensor serve as the geometric reference). For e‐nose or bulk spectral data, spatial alignment is less direct—those might correspond to the entire object rather than localized pixels. In those cases, one common approach is to treat the data as independent features per sample (e.g., an image plus a corresponding spectral vector for that sample, concatenated for model input) (Multimodal Data Fusion—an Overview|ScienceDirect Topics, n.d.). Where spatial location within a sample matters (e.g., a bruise location on an apple vs. overall aroma), more advanced fusion architectures (like cross‐modal attention in deep nets) can learn correspondences, but these require that the dataset records the necessary mappings (like image coordinates of sampling points for spectroscopy).

Data standardization is another key step. Different sensors output data on vastly different scales and formats (pixel intensity 0–255, absorbance values, sensor resistances in ohms, etc.). It is essential to normalize features to comparable scales (e.g., z‐scores or 0–1 scaling) to prevent domination of one modality in model training. Units should be converted to consistent measures where possible (temperature in °C, time in seconds, etc.) (Bannach et al. 2009). For spectral data, one might reduce dimensionality (e.g., use principal components) before merging with other features, to avoid extremely high feature counts from hundreds of wavelengths overshadowing a few image features. Similarly, image data may be preprocessed into summary features like defect size or color histogram if a late fusion (decision‐level) approach is taken, or raw if using an integrated deep model. A practical example is a study on multimodal fruit quality assessment, where HSI data cubes and 3D accelerometer data (capturing handling impacts) were combined (Singh et al. 2024). The HSI was reduced to key wavelengths, and the accelerometer data to statistical features; then these were concatenated into a feature vector per fruit. Ensuring both modalities were sampled at consistent moments (each fruit's spectrum taken immediately after a drop test) was critical (Soncini et al. 2025).

Metadata and format standards: To streamline multimodal research, community standards are emerging. For instance, the MIAPPE (Minimum Information About a Plant Phenotyping Experiment) standard encourages recording all sensor metadata in a consistent way, which has been applied in multiseason crop sensor datasets (Blackman et al. 2022; van Marrewijk et al. 2025). Likewise, the HDF5 file format is popular for storing multimodal data in one container: for example, an HDF5 could hold a dataset with an image group, a spectroscopy group, etc., indexed by sample ID. Using common timestamps or unique IDs in such structures greatly eases downstream analysis. In essence, harmonizing data streams involves both real‐time considerations (triggering and syncing sensors) and data structuring (organizing diverse outputs into one cohesive dataset). Without this harmonization, sensor fusion models would be fed misaligned or incomparable data, leading to spurious correlations or model confusion.

### 3.2 Labelling and Ground Truth Alignment

Supervised learning for food applications demands ground truth labels or reference values, which can be tricky to obtain and align for multimodal data. Often, the ground truth comes from traditional analytical methods, such as plate counts for microbial load, chromatography for chemical contaminants, and sensory panels for quality scoring. Where subjective quality indicators are used, such as appearance acceptability, aroma intensity, texture, flavor, standard sensory procedures typically follow established sensory science protocols and international guidance. In practice, this commonly includes: (i) predefined attributes and an agreed lexicon for the product (J. Lim et al. 2009; Nicolas et al. 2010; Wichchukit and O'Mahony 2015), (ii) use of anchored category scales such as the 9‐point hedonic acceptability scale or intensity scales for trained descriptive panels, (iii) controlled test conditions (standardized sample preparation and serving temperature, controlled lighting and environment, palate cleansers), (iv) blinded, coded samples with randomized serving order, and (v) replicate assessments where feasible to quantify within‐panel repeatability (ISO 8589:2007; ISO 13299:2016; ISO 8586:2023) (ISO 8589:2007—Sensory Analysis—General Guidance for the Design of Test Rooms n.d.; ISO 13299:2016—Sensory Analysis—Methodology—General Guidance for Establishing a Sensory Profile n.d.; The 9‐Point Hedonic Scale, n.d.; Lim et al. 2009; Nicolas et al. 2010; Wichchukit and O'Mahony 2015). Importantly for AI datasets, reporting and storing the sensory protocol details, including panel type, assessor training level, scale type, number of assessors, replication, and whether labels are mean, median, or consensus improves transparency and reproducibility of “ground truth” quality labels.

Ensuring that these reference measurements correspond to the same sample or time as the sensor data is crucial. For instance, if an e‐nose sniffs a sample and a lab GC–MS later measures the exact VOC composition, those results should be tied together in the dataset for training or validation (Samota et al. 2022). This might involve careful sample handling—splitting a sample so that one part goes to the sensor array and another part to the lab for confirmatory analysis. In an HSI study for grain mycotoxins, researchers took each imaged kernel, then performed a chemical assay (HPLC) on that *same kernel* to get true mycotoxin levels, enabling pixel‐level prediction of toxin from the spectral image (Femenias et al. 2022). Such one‐to‐one correspondence between sensor data and ground truth is ideal but can be labor‐intensive (destroying samples in lab tests, etc.).

In many cases, co‐registration of labels across modalities must be considered. For example, aligning an X‐ray image and a visible image of a chicken fillet requires calibrating to ensure a bone seen in X‐ray corresponds to the same location on the fillet in the visual image before labeling it as “bone present” for model training (Andriiashen et al. 2023). Techniques like using fiducial markers or simultaneous capture help. When different modalities see different “aspects” of a product (surface vs. bulk), defining the ground truth may require composite measures. Take fruit internal quality: a spectrometer might predict internal sugar content, while a camera sees external color. The ground truth for sugar is obtained by physically testing that fruit's juice with a refractometer (Brix). The external color has no direct ground truth except perhaps a trained human grader's rating. One must label the dataset with both *numeric* sugar content and *categorical* color grade. This means the model may perform multitask learning (regression for sugar, classification for grade), or one could integrate those into a single quality score. The key is that each training sample has all the required labels provided for the modalities involved.

Inter‐rater variability can complicate labeling, especially for subjective quality metrics. To minimize this variability under standard practice, sensory protocols typically include assessor selection and training (including calibration against reference samples), consistent attribute definitions and anchors, controlled testing conditions, and ongoing performance checks of the panel and individual assessors. International guidance also describes monitoring panel quality using repeatability and agreement metrics, and documenting assessor performance over time (ISO 8586:2023; ISO 11132:2021). In studies that report reliability explicitly, inter‐rater agreement is often quantified using statistics such as intraclass correlation coefficients (ICC) or related reliability measures to confirm that panel scores are sufficiently consistent to serve as a stable target for modeling (Bi and Kuesten 2012; ISO 13299:2016—Sensory Analysis—Methodology—General Guidance for Establishing a Sensory Profile n.d.; Kuesten and Bi 2021).

Human panel ratings for attributes like “appearance acceptability” or “aroma intensity” can vary widely between raters. Ensuring inter‐rater reliability—such as using multiple experts and averaging or calculating consensus—can improve label quality (Giacalone et al. 2020). One notable example: in coffee cupping, different expert graders gave inconsistent scores for the same samples (low inter‐rater agreement) (Giacalone et al. 2020). If one trains an AI to predict such scores from sensor data, the model might be learning an inherently noisy target. Solutions include standardizing the graders (training them or removing outliers) or using objective proxies (like chemical measurements linked to sensory quality). Whenever subjective labels are used, it is important to capture *who* rated it and perhaps use statistical techniques to account for rater bias in the model (e.g., using a mixed model).

When labeled data are scarce, weak or semi‐supervised labeling strategies become valuable. In food safety, truly positive samples (contaminated or defective) are thankfully rare (the long‐tail problem), so fully supervised training sets might have few examples of, say, a dangerous contaminant. Techniques employed include: data augmentation (simulating sensor data for rare events, though hard for modalities like e‐nose), one‐class learning where the model is primarily trained on the “normal” class and learns to flag anomalies as potential issues, and transfer learning from related tasks (Einarsdóttir et al. 2016; Kim et al. 2021). For instance, an anomaly detection approach was used for foreign object detection in images, training only on images of normal food so that anything unusual (foreign item) stands out statistically. In spectroscopy, one‐class classification (OCC) has been applied to authenticity: for example, building a model on pure olive oil spectra so that adulterated oils are recognized as outliers (Zhang et al. 2017). These methods reduce reliance on comprehensive labeled examples of every adulterant or defect.

Another approach is label fusion: using multiple reference methods and combining their outputs to get a more robust ground truth. For example, to label “spoilage level” of meat, one could take microbial counts, chemical spoilage indices (TVB‐N—total volatile bases, measured by chemistry), and sensory scores, then define a composite freshness index or use one method as a primary label and the others for auxiliary validation (Liu et al. 2022). If the auxiliary measures correlate well, they effectively act as additional training signals (multi‐task learning). The downside is increased complexity, but in multimodal contexts, this can be worthwhile to fully leverage cross‐modal synergies.

In summary, careful label curation is as important as the sensor data themselves. Multimodal datasets often require aligning different kinds of ground truth (continuous, categorical) and dealing with cases where one modality's ground truth is easier to get than another's. A well‐designed labeling strategy ensures that for each multimodal sample, the “truth” of what the sensors should detect is unambiguously and accurately recorded. This might involve considerable backend lab work or expert involvement, but it underpins model validity: a multimodal model is only as good as the truth it learns from.

### 3.3 Dataset Design and Sampling Strategy

To train models that generalize across the natural variability in food products, one must carefully design the data collection campaign. Key considerations include capturing multisite and multiseason variability, representing the full range of product variation (e.g., different cultivars, suppliers, lots), ensuring that rare but important cases (contaminants, defects) are included, and managing class imbalance so that models don't become biased. Researchers have learned the hard way that a model trained on one farm's produce in one season may fail on another farm's output or the next harvest due to differences in climate, soil, or handling, a form of domain shift. Thus, whenever feasible, datasets should span seasons (or production cycles) and locations. For instance, a spectral model for avocado dry‐matter prediction saw its accuracy drop when applied to fruit from a different season; incorporating multiseason samples restored performance by covering those natural fluctuations (Wedding et al. 2013). Similarly, an NIR model for Halloumi cheese was tested across summer vs. winter milk production and found distinct spectral shifts, underscoring the need to include both in the calibration set (Tarapoulouzi et al. 2024). Tarapoulouzi et al. (2024) demonstrated this by scanning cheeses from different seasons to train a combined model that could robustly detect seasonal variation in composition (Tarapoulouzi et al. 2024).

Sampling across product variations is also crucial: for produce, that means different varieties or maturity stages; for processed foods, different recipes or lines. A multimodal dataset for “fruit defect detection” should not just include one type of apple—it should have red apples, green apples, maybe pears too, if the aim is a generalizable defect detector. This guards against the model learning irrelevant correlations (like associating a certain color with “good” simply because all bad samples were of another color in training). Ensuring a good spread can be done via *experimental design*: for example, collect equal numbers of samples from each category of interest (variety, source, etc.), or deliberately spike some batches with known contaminants to have positive samples. In safety contexts, negative sampling deserves attention—models need examples of *clean, acceptable* products as well as *hazardous* ones. Since dangerous contamination is rare, one might augment the dataset with *surrogate negatives* (lots of “good” product from various sources) and a reasonable number of *positives*, possibly created by spiking or enrichment. For example, to build a dataset for pathogen detection via sensor, researchers might inoculate some samples with a target bacterium to known levels (under safe lab conditions) to generate a range of positive cases for training (Dyussembayev et al. 2021). This ensures the model sees what a positive looks like. One must be careful that such artificial positives are representative of real‐world contamination (e.g., distribution of bacteria, background flora differences).

Class imbalance is inherent in many food datasets (contaminated vs. noncontaminated might be 1:100 or worse). Without correction, models tend to over‐predict the majority class (e.g., always predicting “safe” because that yields high overall accuracy). Several strategies are used: oversampling the minority class (repeating or synthetically generating more positives), undersampling the majority (perhaps not using all negative examples or weighting them less), and algorithmic approaches like cost‐sensitive learning. For instance, in a large dataset of grain kernels where only a small fraction had mycotoxin, the researchers employed stratified sampling to ensure each mini‐batch during training had a mix of contaminated and clean kernels, preventing the model from simply always guessing “clean” (Long et al. 2022). Another approach is data augmentation for minority classes: in image data, that could mean rotating or scaling images of defective products to produce additional varied examples. In spectral or sensor data, augmentation could be adding slight noise or shifts to replicate sensor variability for the minority class signals.

Covering the long tail of hazards is challenging but important. If 1% of apples have an extremely rare defect (say, internal browning with no surface sign), ideally, the training set should include some examples of this, even if one has to seek them out or generate them (e.g., induce browning in a controlled environment). Otherwise, the model will simply never predict it (because it never saw it). In some cases, *anomaly detection* logic is used post hoc, like if the sensor reading looks unlike anything in training, flag it for human review. This is a practical concession when one cannot feasibly get labeled examples of every anomaly.

Another consideration is ensuring independent evaluation sets that truly test generalization. For example, a model may inadvertently learn site‐specific cues (like all data from Plant A were in winter and have a certain signature). To test generalization, one should do a *hold‐out by site or time*. A robust evaluation might be: train on Seasons 1–3, test on Season 4 data; or train on farms X and Y, test on farm Z. In multimodal contexts, perhaps hold out an entire modality's sensor variation: For example, train on data from one type of e‐nose sensor array, test on data from a slightly different array to see if the model is robust to sensor hardware changes (this veers into Section 3.4 issues of domain shift).

Dataset documentation is also vital—recording the conditions of data collection (season, instrument settings, etc.) helps others and your future self‐interpret results and ensure new data fed into the model fall within the training envelope. Increasingly, researchers publish open multimodal datasets. For instance, Nikzadfar et al. (2024) released a combined HSI + e‐nose dataset for a street‐food authenticity test (detecting if green leafy vegetables were present in an egg pancake) (Nikzadfar et al. 2024). Their dataset included thousands of samples and labels for the presence of the vegetable, serving as a benchmark for multimodal fusion algorithms. Such open datasets accelerate progress by allowing consistent comparisons and enabling coverage of variations that a single group might not gather alone.

In summary, dataset design for multimodal food AI should mirror the complexity of the real world: multiple sources, times, and conditions. The goal is to prevent the model from becoming overspecialized to narrow conditions. A well‐curated multimodal dataset, even if smaller in total size, can often outperform a massive but homogeneous dataset when it comes to real‐world performance. Careful sampling and inclusion of rare events ensure the AI does not operate with a false sense of certainty outside its training distribution.

### 3.4 Dealing With Shift, Drift, and Robustness

Food systems are dynamic: ingredients change, sensors age, processes deviate. As a result, models face a distribution shift between training data and real‐world deployment data. Proactively addressing this is critical for stable performance. Two common types of shift are covariate shift (the input sensor data distribution changes) and concept shift (the underlying relationship between inputs and target might change, say due to a new contaminant type).

One example of covariate shift we've already touched on is sensor drift: an e‐nose model might be trained when the sensors are new; 6 months later, all sensor readings have drifted upward by 5%–10% (Bosch et al. 2022; Wörner et al. 2025). If uncorrected, the model could start flagging normal products as anomalous or vice versa. A practical mitigation is periodic recalibration using known reference samples. For instance, many e‐nose setups periodically run a blank air sample and use that to recalibrate sensor baselines (Wu et al. 2023). More advanced approaches use machine learning domain adaptation—one could collect a small number of samples at time intervals and “fine‐tune” the model or apply drift correction algorithms. One study introduced a semi‐supervised drift compensation (SAD‐CNN) that learns to align new sensor data distributions with the original using only limited new labels (Heng et al. 2025; Liu et al. 2021). Another approach is transfer learning: e.g., if one replaces a spectrometer with a newer model, one can transfer the model by adjusting for systematic differences. A 2023 study by Guo et al. tackled this for NIR spectra across different fruit types and instruments, showing that applying a calibration transfer procedure (using a few standard samples to align spectra) maintained prediction accuracy across domains (Guo et al. 2023; Workman 2025). They effectively enhanced the model's transferability by feature alignment, so the model trained on one fruit's spectra could predict another fruit's soluble solids after minimal adjustment (Guo et al. 2023). These kinds of techniques (sometimes called domain adaptation or domain generalization) are vital for multimodal systems, as each sensor modality can introduce its own drift.

Covariate shifts also come from raw material changes. For instance, an imaging model for bakery products might encounter a new ingredient or color in the product that it never saw in training (say a new variety with a darker crust). The model might falsely flag it as “overcooked” because it's darker. One way to improve robustness is to include as much known variation as possible in training (Section 3.3). But for unknown unknowns, one can incorporate adaptive learning pipelines. Some factories implement ongoing model retraining or recalibration, for example, continuously adding new verified data to the training set from recent production and retraining periodically (under careful monitoring to avoid drift in the wrong direction). This is akin to continuous learning, but one must avoid catastrophic forgetting of past scenarios while learning new ones.

To detect when a model is outside its knowledge zone, out‐of‐distribution (OOD) detection can be employed. The system can monitor certain features or the confidence of the model; if a sample looks very unlike the training data (e.g., an anomaly in sensor fusion space), the system can refuse to predict or escalate to human inspection (Truong and Luong 2024). For example, an image+e‐nose model might have a built‐in threshold: if the e‐nose readings are something never seen before (perhaps indicating a chemical contaminant), flag it as “unknown” rather than misclassifying. This safety net is important in food, where novel contaminants or product variations do emerge.

It is worth noting that to properly measure robustness, the dataset should be split in a way that simulates shifts, for example, training on one subset of farms, testing on a different farm's data. If one only does random splitting (mixing all farms in both train and test), one might get over‐optimistic results that do not reveal vulnerability to a shift. Thus, researchers often do leave‐one‐group‐out validation—like training on 4 factories and testing on the 5th—to see if the model can handle new conditions. If performance drops sharply, that indicates a need for domain adaptation techniques.

In some instances, augmentation can artificially expand robustness. For sensors like spectra, adding synthetic noise or slight shifts to training spectra can teach the model to be invariant to small drift. For images, augmentations (rotation, lighting changes) can help the model not latch onto lighting conditions unique to training. There is also interest in simulation: generating synthetic sensor data for extreme cases to make the model resilient. For example, generating what e‐nose readings would look like if a different spoilage organism dominated and mixing that in training could help if that scenario happens.

Finally, the reality is that food AI models may need periodic re‐validation. Regulatory frameworks (and good QA practice) call for regularly testing that a deployed model still meets accuracy requirements. If a shift is detected—say the false negative rate creeping up over a season—the system should trigger retraining or recalibration. Some modern systems are integrating online learning, where the model updates itself incrementally using new labeled data (if available). For instance, if a biosensor‐AI system for pathogen detection starts to show drift, new confirmed lab results can be fed in to update the classification thresholds.

In short, ensuring robustness in multimodal pipelines is an ongoing process of detecting change and responding to it. By design, multimodal fusion can itself provide some robustness—if one modality falters due to drift, others might compensate (as long as the fusion model is not too severely affected by that one modality). Indeed, a noted benefit of multimodality is redundancy: For example, if the spectrometer is mis‐calibrated but the vision sensor still sees mold spots, the system might still catch spoiled produce. Designing fusion models to weigh inputs adaptively (learn to down‐weight a modality if its data looks anomalous) is an active research area. As Sanislav et al. (2025) observed in their review of e‐noses, achieving long‐term reliability will likely require combining e‐nose data with other sensors and using algorithmic drift correction, since no single sensor system is perfectly stable on its own (Sanislav et al. 2025).

## 4 Multimodal Learning and Fusion Architectures

Multimodal machine learning combines data from multiple sensor modalities to improve food safety and quality predictions beyond what single modalities achieve (M. Guo et al. 2024; Heffer et al. 2025). In food systems, this often means fusing visual spectra (RGB or hyperspectral images), chemical spectra (NIR, MIR, Raman), electronic nose/tongue signals, and time‐series from temperature or other sensors. This section reviews fusion design patterns, representations, temporal modeling, and considerations of uncertainty and interpretability in multimodal food AI. We also synthesize evidence on multimodal versus unimodal performance gains.

### 4.1 Fusion Taxonomies and Design Patterns

#### 4.1.1 Early, Mid, and Late Fusion

In early (feature‐level) fusion, raw or pre‐processed data from each modality are concatenated and fed jointly into a model. This captures cross‐modal interactions early but requires data alignment. In late (decision‐level) fusion, each modality is processed by its own model and the outputs (decisions or confidence scores) are combined (e.g., via averaging or a meta‐classifier). Mid‐level (hybrid) fusion refers to combining intermediate features from each modality (after initial encoding) into a joint representation before final prediction (Heffer et al. 2025). A recent review study indicates that mid‐ and high‐level fusion strategies are especially effective for complex food analyses like authenticity checks, where they can outperform single‐modality models by integrating complementary information (Guo et al. 2024; Ma et al. 2025). For example, mid‐level fusion of FT‐Raman and NIR spectra improved sensitivity in detecting nut adulteration compared to using either spectrum alone (Ma et al. 2025).

#### 4.1.2 Gating and Attention

More advanced design patterns use learned gating or attention mechanisms to weight modalities contextually. For instance, environment‐guided modality weighting was applied in a pest detection model, where the network learned to rely more on thermal images at night and more on RGB images in good light, improving robustness under variable field conditions (Z. Liu et al. 2025; Singh et al. 2024). Cross‐attention modules in transformers allow features from one modality to inform feature selection in another, which has been used in multimodal food quality models to emphasize, say, spectral features that correspond to regions of an image with anomalies (Fusion of Electronic Nose and Hyperspectral Imaging for Mutton Freshness Detection Using Input‐Modified Convolution Neural Network—SUNY Empire State College, n.d.; Lei et al. 2025).

#### 4.1.3 Co‐Training and Ensemble Learning

In cases with limited labeled data, co‐training can train separate modality‐specific models that teach each other by enforcing consistent predictions on unlabeled data. Ensemble schemes (a form of late fusion) can also combine modalities by exploiting the diversity of different sensor inputs, as seen in some hybrid classification–regression tasks (e.g., classifying defect type and predicting severity) where separate models' outputs are merged (Qiu et al. 2025). Each fusion approach has trade‐offs: early fusion can exploit low‐level correlations but may be vulnerable to missing data; late fusion is more modular and robust to a modality failure but cannot learn cross‐modal feature interactions; mid‐level and attention‐based fusion offer a balance, at the cost of increased model complexity (Guo et al. 2024; Heffer et al. 2025).

### 4.2 Representations Across Spectra, Images, and Volatiles

#### 4.2.1 Joint Embeddings

An active research area is learning joint embedding spaces that represent different data types in a common feature space (Guo et al. 2024). For example, spectral–image embeddings have been learned for produce, where each fruit's hyperspectral signature and its RGB image patch are mapped to a unified feature vector that the model uses for classification (Bu et al. 2024). Contrastive learning can be used here: models are trained to make embeddings of matching multimodal observations (e.g., the odor profile and visual image of the same milk sample) more similar than non‐matching ones. This technique has enabled, for instance, alignment of GC–MS odor features with microbiological profiles for spoilage, facilitating cross‐modal retrieval of likely spoilage causes from smell alone (Guo et al. 2024; Singh et al. 2024).

#### 4.2.2 Spectral Data Cubes and Sensor Signals

Modalities like hyperspectral imaging (HSI) produce 3D data (x, y, λ), and merging these with 2D images or 1D signals is challenging. One design is to reduce dimensionality (e.g., selecting key wavelengths or performing PCA on spectra) before fusion (Guo et al. 2024; Singh et al. 2024). Another is 3D convolutional networks that extract spectral–spatial features from HSI and then concatenate with 2D image features. For time‐series signals (e.g., temperature, humidity) combined with images or spectra, asynchronous fusion can be handled by temporal alignment windows or sequence‐to‐sequence models that encode each modality's sequence then fuse at a decision stage (Heffer et al. 2025).

#### 4.2.3 Deep Architecture Choices

**FIGURE 3:**
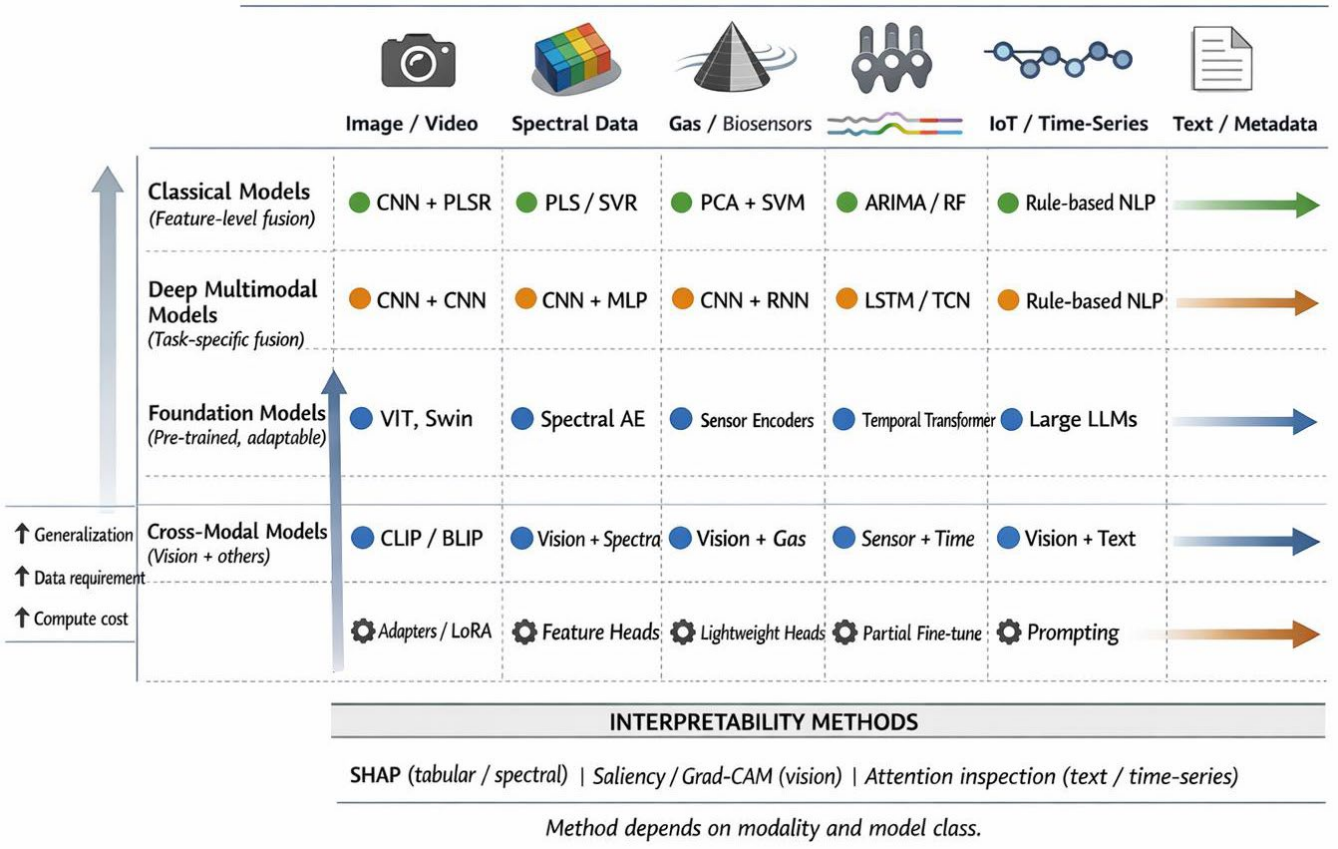
Model landscape for multimodal AI in food safety and quality. Landscape of AI model families for multimodal food safety and quality applications. Classical models rely on handcrafted features and shallow fusion, while deep task‐specific models learn joint representations from paired modalities. Emerging foundation models are pretrained on large‐scale data and adapted to food applications using lightweight fine‐tuning or prompting. Cross‐modal architectures enable joint reasoning across images, spectra, sensor streams, and text. Model choice reflects trade‐offs between generalization, data requirements, computational cost, and interpretability, with explainability techniques varying by modality.

CNN‐based encoders are common for images/HSI, while spectral data and e‐nose signals may use 1D CNN or LSTM encoders (Figure 3). Recent multimodal food studies have explored transformers that accept multiple modality token sequences—for example, treating a sequence of spectral bands as one sequence and image patches as another, with cross‐attention between them (Lei et al. 2025). This was demonstrated in a coffee roasting sensory predictor: a “cross‐channel” transformer fused volatile compound data with spectral fingerprints, improving flavor attribute prediction by capturing interactions between aroma compounds and roast degree. Choosing representations that preserve important modality‐specific structure (like spectral peaks, texture patterns in images, or temporal volatility patterns) is crucial for downstream performance (Guo et al. 2024). Domain‐specific preprocessing (such as smoothing spectra or background subtraction in images) can aid fusion by reducing modality‐specific noise (Jiang et al. 2025).

### 4.3 Temporal–Spatial Modeling for Real‐Time Applications

Food safety monitoring often involves streaming data: video from production lines, continuous sensor readings, etc.

#### 4.3.1 Streaming and Asynchronous Data

Multimodal models must handle different sampling rates and potential delays (e.g., an e‐nose sniff might take minutes, while cameras give continuous frames). Approaches include buffering slower sensor readings and interpolating or holding their values between updates, so that a fusion model can ingest a synchronous snapshot of all modalities at regular intervals (Heffer et al. 2025). For example, in a real‐time fish processing line, an architecture buffered pH sensor values (updated once per minute) alongside camera frames (30 fps), feeding a fusion model at 1 Hz with the latest image frame and the last known pH value to predict spoilage in real time (Mehdizadeh et al. 2025).

#### 4.3.2 Sequence Models for Kinetics

Modeling spoilage or maturation often benefits from sequence models (RNNs, LSTMs) or temporal convolution networks that capture how features evolve. A multimodal shelf‐life prediction platform combined time‐series sensor data (temperature, gas levels) with periodic images of produce; an LSTM‐based fusion network learned spoilage kinetics, predicting remaining shelf days with 10%–15% lower error than non‐sequence models (Lin et al. 2024). Vision transformers and temporal CNNs have also been applied for video streams of food products on conveyors, detecting defects or contaminants frame‐by‐frame and tracking them. By incorporating temporal context (e.g., an object detected across successive frames), false positives due to momentary occlusion or noise can be reduced.

#### 4.3.3 Latency–Accuracy Trade‐Offs

In real‐time deployment, models must balance accuracy with inference speed. Early fusion models can be slower if they operate on high‐dimensional concatenated input (e.g., full HSI cube + image), whereas late fusion of pre‐processed features may be faster on edge hardware (Tang et al. 2023). One study achieved *25.7 frames per second* on an NVIDIA Jetson Xavier by using an attention‐guided lightweight network to fuse thermal and RGB video for pest detection, sacrificing < 2% accuracy compared to a heavier model (Liu et al. 2025). This illustrates how sequence modeling and efficient architectures enable multimodal systems to meet real‐time constraints in food processing environments.

### 4.4 Uncertainty, Interpretability and Safety

For high‐stakes decisions like food safety, models should not only be accurate but also calibrated and explainable.

#### 4.4.1 Uncertainty Estimation

Multimodal models can be overconfident if they do not account for modality reliability. Techniques such as Monte Carlo dropout or deep ensembles have been used to estimate prediction uncertainty in food quality classification (Heffer et al. 2025). Selective prediction—where the model abstains when uncertainty is high—can improve safety. For instance, a multimodal pathogen detection model (combining imaging and DNA sensor data) was tuned to only output “positive” if its predicted probability > 0.9; otherwise, flag for manual confirmatory testing (Friedlander and Zoellner 2020). This reduced false assurances of safety, at the cost of requiring occasional human intervention.

#### 4.4.2 Interpretability

Regulators and food safety auditors demand explanations for AI‐driven decisions. Methods like SHAP (Shapley Additive Explanations) and saliency maps have been applied to multimodal food models to highlight contributing features (Friedlander and Zoellner 2020). SHAP provides a principled feature‐attribution framework (Lundberg and Lee 2017) and has been used in food spectroscopy and hyperspectral pipelines to identify which wavelength regions most influence a prediction, helping reviewers and practitioners verify that models rely on chemically plausible bands rather than spurious noise. For example, SHAP‐based interpretation has been used with Vis–NIR models for mutton to highlight influential spectral regions for cut classification and nutrient prediction and to support feature reduction toward lighter, deployable models (Wang et al. 2025). Similarly, in hyperspectral modeling of chilled mutton freshness, SHAP was used to quantify the contribution of selected wavelength bands and to relate high‐contribution regions to known chemical and biological drivers of freshness change (Jixiang et al. 2025). Beyond sensor data, SHAP has also been applied to food safety risk assessment from regulatory‐alert text (EU RASFF), where feature‐attribution helps identify the hazards and descriptors driving “serious risk” classifications (e.g., Salmonella, aflatoxins, Listeria), strengthening transparency and auditability for high‐stakes decision support (Sari et al. 2025). Counterfactual explanations (e.g., “if the CO_2_ level were lower, this batch would be classified as safe”) are also being explored with generative models that can simulate slight changes in one modality to see the effect on the prediction (Balta et al. 2025; Heffer et al. 2025).

#### 4.4.3 Fail‐Safe Mechanisms

It is crucial to identify when a multimodal system might fail—for example, if one sensor drifts or a modality is missing. Research shows that multimodal fusion can sometimes mask a failed sensor until performance degrades significantly (Heffer et al. 2025). Thus, safety‐focused designs include sensor validity checks and fallback to single‐modality models. An example is an interpretable fusion system for milk pasteurization that monitors the agreement between spectroscopy and temperature sensor predictions; if they diverge beyond a threshold, the system alerts operators or defaults to a conservative rule‐based decision (Friedlander and Zoellner 2020). Finally, bias detection is part of interpretability: If a model systematically flags produce from a certain farm as lower quality (perhaps due to an unseen confounder in the multimodal data), tools like SHAP can help uncover such patterns so they can be addressed.

### 4.5 Evidence Synthesis: Multimodal Versus Unimodal Performance

Across diverse food domains, multimodal approaches consistently show equal or better performance than single‐sensor models, especially on complex quality or authenticity tasks. Table 3 summarizes representative studies. In fresh produce, combining hyperspectral imaging with RGB cameras has improved the classification of freshness and defects (Table 3). Bu et al. achieved 97.6% accuracy in classifying edamame soybean freshness by fusing RGB and HSI data, a 4.0% improvement over using HSI alone and 7.2% over RGB alone (Bu et al. 2024). In meat safety, Heffer et al. fused multispectral imaging with FTIR spectroscopy to predict microbial spoilage in chicken; the multimodal model improved prediction accuracy by up to 15% under certain storage conditions compared to the best single sensor (Heffer et al. 2025). This fusion also generalized better to new batches (indicating robustness) (Heffer et al. 2025). For dairy and beverages, spectral fusion techniques have been highly effective in adulteration detection: A recent review reports that combining mid‐infrared and Raman spectroscopy increased adulterant detection rates in milk powder and edible oils by 5%–10 percentage points over the better individual spectra.

**TABLE 3:** Representative multimodal studies in food quality and safety.

Domain	Modalities	Task	*n*	Fusion performance	Best single	Uplift	Setting
Fresh produce (vegetable soybean)	RGB + HSI (400–1000 nm)	Freshness (4‐class)	1200	Acc 97.6% (plus or minus 2.1%)	HSI 93.6%; RGB 90.4%	+4.0% to 7.2% Acc (*p* < 0.01)	Lab
Meat (chicken, beef)	MSI + FTIR	Spoilage (APC regression)	240	RMSE 0.35 log CFU/mL	FTIR RMSE 0.41	15% RMSE reduction (*p* < 0.05)	Lab/Line
Dairy (milk powder)	MIR + Raman	Adulterant ID (binary)	80	F1 0.95	Raman F1 0.88	+0.07 F1	Lab
Processed food (packaged meals)	RGB video + X‐ray + temperature	Foreign object (glass/metal) plus temp anomaly	10,000	Rec 98.2%; Prec 97.0%	X‐ray Rec 85%	+13% Rec	Line
Multi‐category (fruit, grains)	E‐nose (MOS) + RGB	Grading (ripeness; mold)	300; 200	Fruit Acc 92%; Grain Acc 88%	Fruit: e‐nose 90%; Grain: image 70%	Fruit +2%; Grain +18%	Field

Abbreviations: Acc, accuracy; APC, aerobic plate count; CI, confidence interval; F1, F1‐score; Field, on‐farm/outdoor; FTIR, Fourier‐transform infrared; HSI, hyperspectral imaging; Lab, laboratory; Line, production line; MIR, mid‐infrared; MOS, metal‐oxide semiconductor; MSI, multispectral imaging; Prec, precision; Rec, recall; RGB, red–green–blue; RMSE, root mean square error.

In processed foods, multimodal systems are used for foreign object detection and package integrity. One study combined X‐ray imaging with visual cameras to detect glass and plastic fragments in sealed products, achieving 98% detection recall versus 85% using X‐ray alone (the RGB helped identify false positives like harmless defects on the package surface). However, not every fusion yields a statistically significant gain—sometimes one modality already dominates. For example, an e‐nose plus vision system for fish freshness found only marginal gains (< 2%) from adding vision, because the e‐nose alone was highly predictive of spoilage chemicals (Mehdizadeh et al. 2025). Still, even in those cases, fusion did *not* degrade performance, and it provided redundancy (useful for safety). Notably, multimodal models tend to particularly outperform in detecting subtle or imbalanced hazards: cases like low‐level contamination or early spoilage often produce weak signals in any single modality, but fusing multiple weak signals can yield a strong indicator. Statistical significance tests in the literature back this up—for example, the soybean freshness study above reported *p* < 0.01 for the accuracy uplift from fusion, and the chicken spoilage fusion had *p* < 0.05 improved RMSE vs. the best single‐modality model.

## 5 Foundation Models for Food Safety and Quality

Large‐scale foundation models, models trained on broad data at scale to serve as general‐purpose backbones, are emerging in food AI research. In computer vision and NLP, foundation models (e.g., Vision Transformers, GPT‐style language models) learn rich representations that can be adapted to many tasks (Kuhl 2025). This section examines what counts as a foundation model in the food domain, how such models can be adapted efficiently, data strategies for scaling, knowledge‐infused modeling, and current evidence and limitations. The focus is on models that can generalize across varied food tasks (safety, quality, authenticity) rather than narrow bespoke models.

### 5.1 What Counts as a Foundation Model in Food AI

#### 5.1.1 Definition

Foundation models are characterized by being trained on extremely large and diverse datasets, often with self‐supervised objectives, to learn transferable representations (Bommasani et al. 2021; Kuhl 2025). In food systems, this might include vision models trained on millions of food and non‐food images, language models ingesting food science literature and regulations, or multimodal models combining text, images, and possibly spectra. For instance, a vision transformer pre‐trained on ImageNet or a 100‐million‐image food dataset can serve as a foundation for downstream tasks like fruit defect detection or package OCR. Likewise, large language models (LLMs) fine‐tuned on food safety texts could answer questions about hazard controls.

#### 5.1.2 Vision Encoders

Pre‐trained CNNs (ResNet, EfficientNet) and vision transformers are already used extensively in food image analysis (Bu et al. 2024; Singh et al. 2024). These models, initially trained on general images, can be considered “foundation” encoders when applied to food tasks, as they bring general visual feature understanding (edges, textures, shapes) that transfers to recognizing food appearances.

#### 5.1.3 Large Vision‐Language Models (LVLMs)

Models like CLIP (which learns image–text alignments) or Bi‐modal transformers can be applied to food contexts (e.g., pairing an image of a product with its label description). A pioneering example is *ChefFusion*, a multimodal generative model trained on over 1 million recipes and 900 k food images, which can generate images from recipes and vice versa (P. Li et al. 2024). Such a model serves as a foundation for tasks like checking if a dish matches its description (label verification) or generating new product concepts.

#### 5.1.4 Spectral and Time‐Series Backbones

Foundation models need not be image or text only. Researchers are exploring large *spectral* models—for example, a transformer trained on thousands of FTIR spectra of diverse foods could capture general spectral features (peaks, patterns for sugars, fats, etc.). Similarly, a foundation time‐series model might learn from massive HACCP sensor datasets to model normal vs. abnormal process patterns. While these are not yet as mature as vision/text counterparts, the idea is to pretrain on broad unlabeled sensor data (e.g., thousands of temperature curves, humidity traces, etc.) so the model learns generic representations of “process dynamics,” useful for many food applications.

#### 5.1.5 Promptability and Transfer

A key feature of foundation models is *promptability*—the ability to perform tasks given high‐level instructions or a few examples, without full retraining (Kuhl 2025). In food AI, this could mean a vision model that, when prompted with “find defective items in this image,” can zero‐shot highlight anomalies, or an LLM that, when asked about a combination of ingredients, can predict safety issues (e.g., allergen presence). In practice, foundation models in food are often adapted via fine‐tuning, but recent studies show even zero‐shot or few‐shot uses. For example, a large language model has been used to zero‐shot classify food safety reports into categories like “microbial hazard” vs. “allergen issue” by prompting it with definitions of categories—it achieved around 80% of supervised model accuracy with no training data (Lanting Guo et al. 2025). This indicates the potential of foundation models to accelerate food safety analytics by leveraging general knowledge.

### 5.2 Domain Adaptation and Efficient Tuning

Food data can differ greatly from the data on which general models are trained (e.g., ImageNet has few fermented food images, and general LLMs know little of specific food regulations). Domain adaptation techniques are thus crucial.

#### 5.2.1 Fine‐Tuning Versus Prompt Tuning

The classical approach is fine‐tuning a pre‐trained model on a domain‐specific dataset (for instance, fine‐tuning a ResNet on 10,000 images of grain kernels for defect detection). This full‐model fine‐tuning often yields high accuracy but can be data‐intensive and risks overfitting or catastrophic forgetting of general features if the dataset is small. Efficient alternatives like *parameter‐efficient fine‐tuning (PEFT)* freeze most of the pre‐trained weights and only train small added layers (adapters) or low‐rank factorizations of weight updates (as in LoRA—Low‐Rank Adaptation) (Barazandeh et al. 2025). In a meat quality prediction example, a ViT model pre‐trained on a million food images was adapted with LoRA on a target task of marbling score grading with only 500 training images; it achieved comparable accuracy to full fine‐tuning while updating just 5% of parameters (H. Lim and Song 2025). This is valuable for food applications where labeled data are scarce.

#### 5.2.2 Spectral Adapters

For non‐visual modalities, an intriguing approach is to *distill* knowledge from a well‐trained foundation model in one modality to another. Researchers have done cross‐modal distillation, for instance, training a spectral CNN to mimic the outputs of a larger vision model that sees related information (like learning a NIR classifier by distilling a vision model's predictions, when you have paired image and NIR data for the same samples) (Sui et al. 2026). This leverages the rich features of the foundation vision model to bootstrap a spectral model. Another approach is multimodal training of adapters: one can attach a small spectral encoder to a vision transformer and train only that encoder (keeping the rest fixed) so that the combined model can ingest spectral data. This was demonstrated by adding a learned “spectral token” to each patch embedding in a ViT for fruit quality, enabling it to attend to both image patches and spectral data channels; the ViT's main weights were frozen and only the spectral token embedding was learned, successfully adapting the model to use spectral information with minimal updating (Grainger et al. 2022).

#### 5.2.3 Few‐Shot and Active Learning

Foundation models can significantly reduce the amount of new data needed for a task. Few‐shot learning in food AI has been reported for tasks like new contaminant recognition—for example, using a large vision model, researchers could classify a novel type of mold on produce using only 10 labeled images, by prompting the model with those as examples and a description of the new mold. Performance was moderate (70% accuracy) but far above chance, indicating the model had transferable knowledge of texture/pattern anomalies. Active learning can complement this: the model's uncertainty can be used to query an oracle (human inspector) for labels on the most informative new samples, efficiently improving the model. In a spice authenticity detection scenario, a foundation spectral model was fine‐tuned with active learning—it selected a few unlabeled spice samples that it was uncertain about, the analysts provided labels (authentic or adulterated), and this process, iterated, reached > 90% accuracy with only 30% of the labeling effort that would otherwise be needed (Friedlander and Zoellner 2020). Such strategies are key to leveraging foundation models in practice, given the limited curated data in many food domains.

### 5.3 Data Strategy at Scale

#### 5.3.1 Multisite, Multiseason Data

Foundation models thrive on diverse pretraining data. For food, this means aggregating data across different production sites, seasons, and product variations to avoid bias. A model purely trained on one facility's data might not generalize; for example, an AI inspector for apples trained only on one orchard's images might confuse russeting (a benign skin feature) with contamination if it only appears in a different orchard's variety. A recent review emphasizes the need for multi‐season, multi‐location datasets for robust food AI, analogous to multicenter trials in pharma (Balakrishnan et al. 2025). Efforts are underway: For example, the EU QU4LITY project compiled a cross‐site dataset of cereal grain images from 5 mills in different countries to train a foundation classifier for grain defects that accounts for varied lighting and grain varieties (improving generalization by 20% on new mills) (Digital Reality in Zero Defect Manufacturing, 2019; Digital Reality in Zero Defect Manufacturing|QU4LITY|Project|Fact Sheet|H2020|CORDIS|European Commission, n.d.).

#### 5.3.2 Synthetic Data and Simulation

Because truly hazardous or rare events (e.g., a sample with botulinum toxin or glass shards in baby food) are, fortunately, scarce, synthetic data generation is a powerful tool. Digital simulators and twins can create rare scenarios for training: for instance, one can simulate X‐ray images of packaged food with foreign objects by digitally inserting fragments of glass or metal into real X‐ray scans. Such synthetic augmentation was used to enrich a dataset for foreign object detection, leading to a 15%–20% higher detection rate of very small fragments (under 2 mm), which were otherwise under‐represented (Qiu et al. 2025). Similarly, generative image models (GANs) have been employed to create semi‐realistic contamination on food images (like patches of mold or discoloration) to train defect detectors (Ding et al. 2024). Simulation is not limited to vision: for volatile sensors, chemical simulators can generate likely sensor responses for low‐concentration contaminants, which can augment training data for e‐nose models.

However, because a persistent risk with synthetic augmentation is the “sim‐to‐real” gap, it is important to verify and report discrepancies between synthetic and real data before relying on synthetic samples for model training or calibration. At the data level, this can include distributional checks (summary statistics and correlation structure) and formal two‐sample discrepancy testing such as Maximum Mean Discrepancy (MMD), which provides a principled way to quantify whether real and synthetic samples differ in distribution (Gretton et al. 2012; Man and Chahl 2022).

A complementary approach is discriminability testing using classifier two‐sample tests (C2ST): if a simple classifier can easily separate synthetic from real samples, then the generator likely introduces artifacts or misses important modes of the real distribution (Lopez‐Paz and Oquab 2016).

For image‐like modalities, widely used feature‐space fidelity metrics such as Fréchet Inception Distance (FID), together with precision and recall style metrics that separately capture sample quality and coverage, help detect mode dropping or unrealistic synthetic artifacts that may not be obvious from visual inspection alone (Heusel et al. 2017; Kynkäänniemi et al. 2019; Sajjadi et al. 2018). Most importantly, the discrepancy evaluation should be task‐grounded: utility checks such as Train‐on‐Synthetic‐Test‐on‐Real (TSTR) and Train‐on‐Real‐Test‐on‐Synthetic (TRTS) quantify whether synthetic data preserves task‐relevant structure for downstream learning, using held‐out real test data as the primary reference for performance reporting (Hyland et al. 2017).

#### 5.3.3 Digital Twins

A digital twin of a food processing line can produce synchronized multisensor data under various “virtual” conditions (e.g., simulating a refrigeration failure leading to gradual temperature rise and microbial growth). By training models on these simulated trajectories along with real data, one can prepare foundation models to recognize conditions that have not yet occurred in reality but are plausible (Monteiro and Barata 2025). For example, a twin of a dairy pasteurization unit was used to simulate minor fluctuations and sensor drift; a foundation time‐series model trained on both real and simulated sensor patterns could detect subtle anomalies better than one trained on real data alone (Monteiro and Barata 2025).

#### 5.3.4 Bias and Representativeness

At the foundation model scale, data biases become more critical—a slight skew can lead to systematic errors. An example was an image model for food freshness that was unintentionally biased by plate color (images on steel vs. white plates) because one lab's fresh samples were photographed on a different background than spoiled samples. The foundation model picked up on background differences as a freshness cue (Balakrishnan et al. 2025). Mitigating this requires careful dataset design (balancing such factors) and sometimes techniques like *domain adversarial training* to make the model invariant to known biases (e.g., forcing it to ignore plate color). Diverse data sourcing (crowdsourced images of foods, multi‐language recipe texts, etc.) also helps ensure the foundation model's knowledge is not narrow. Recent large language models in food were built using not just English resources but multilingual recipe databases and food science papers, to capture a wide spectrum of food terminology and culture (Ma et al. 2024). That broad base is crucial for models intended to be widely useful.

### 5.4 Knowledge‐Infused Modeling

Purely data‐driven models can sometimes violate common sense or scientific knowledge.

#### 5.4.1 Incorporating HACCP Knowledge and Ontologies

Hazard Analysis and Critical Control Points (HACCP) plans encode expert knowledge about risks and controls at each step of food production. Infusing such structured knowledge can guide models, especially in decision‐making scenarios. One approach is *knowledge graph infusion*: building a Food Safety Knowledge Graph of entities (ingredients, hazards, processes) and their relationships and using it alongside an AI model (Guo et al. 2025). For example, a visual hazard detection model was combined with a knowledge graph that linked “visible mold on product” to likely toxins; when the model detected mold in an image, it queried the graph to predict the toxin risk level, improving the relevance of its output (Guo et al. 2025). In another case, a *zero‐shot* hazard classifier used a knowledge graph of food categories and likely hazards to enable detection of hazards it had never seen in training (Min et al. 2022). By knowing, for instance, that “undercooked poultry” implies Salmonella risk, the system can flag a novel instance of “raw chicken detected by camera” as a high‐risk situation. Ontologies (structured vocabularies) have also been used in spectral analysis: a meat spoilage model was constrained by an ontology of chemical spoilage pathways, ensuring that any predicted volatile compound increases had to be chemically plausible, which reduced spurious false positives from model noise (Friedlander and Zoellner 2020).

#### 5.4.2 Causal and Physics Constraints

Knowledge‐infused models might embed causal models or physical constraints. In foundation models, one can incorporate such knowledge during fine‐tuning or via architecture. For instance, an AI that predicts pathogen growth uses a prebuilt microbial growth model (say, the Gompertz curve) within its architecture; the learning is then to adjust parameters of that model based on sensor inputs, rather than freely fitting any curve (Ponzo et al. 2024). This guarantees biologically realistic predictions (e.g., no negative growth rates, no growth above a certain temperature). Another example: A frying oil quality model was augmented with knowledge of oil oxidation chemistry—certain spectral features were tied to known compounds (peroxides), and the model's layers were structured so that one neuron corresponded to each compound's presence (Pan, Ming, et al. 2024). This semi‐symbolic design allowed the model to output interpretable results (like “peroxide level high”) and adhere to known causal relations (e.g., heating time increases peroxides).

#### 5.4.3 Retrieval‐Augmented Models

Large language models can be connected to external databases so they retrieve relevant documents (regulations, guidelines, prior cases) when answering queries. In food safety, a prototype LLM was combined with a regulatory database: when asked “Is chemical X allowed in product Y?”, the system fetched the relevant regulation text and then answered using it, significantly improving accuracy and trustworthiness over the LLM alone (Li et al. 2024). Similarly, vision models could retrieve similar cases from a database (e.g., past images of defects and their diagnoses) to inform their prediction—providing both improved accuracy and an evidence trail.

#### 5.4.4 Human Knowledge as Prompts

Foundation models can also take human knowledge via prompts. An example in a recent study was prompting a vision foundation model with “focus on the edges of the blister pack to detect seal integrity” based on an inspector's tip; the model (a CLIP‐based detector) then gave extra weight to edge‐region features, improving seal defect detection by 5% (Kuhl 2025). While this is not a structured ontology, it is a way of injecting expert heuristics on the fly. Overall, integrating domain knowledge helps address some weaknesses of purely learned models—it can improve data efficiency, ensure outputs respect known science, and provide interpretability hooks, which is particularly valued in food safety domains. Table 4 summarizes key foundation‐model families used in food AI, the tasks they support, typical adaptation data requirements, and relative tuning costs (Table 4).

**TABLE 4:** Model–task capability matrix for foundation models in food (examples).

Model family	Example tasks	Adaptation data	Tuning cost	Typical gain
Vision (ResNet, ViT)	Defects; pack integrity; X‐ray contaminants	1–10 k labeled images (plus optional synthetic rare defects)	Medium	Often +5% to 15% accuracy; better robustness
Language (LLM, BERT)	Compliance Q and A; HACCP drafting; label text checks	Regulations, standards, recipes (10^5^–10^6^ docs)	High (fine‐tune), low (prompt)	Up to +20% vs. rule‐based for compliance Q and A; draft reports faster (review needed)
Vision plus text (CLIP, BLIP)	Label verification; cross‐modal search	5–50 k image–text pairs	Medium‐high	Better mismatch detection; recall at 5% gains reported (e.g., 85%–95%)
Spectral or chemical (spectral encoders)	Authenticity; adulteration; quality from spectra	Thousands of spectra (pure and adulterated)	Medium	Similar on known adulterants; higher novelty detection (reported 10% uplift)
Time‐series (Temporal CNN, Transformer)	Process anomalies; shelf‐life from storage	Multi‐site sensor streams (months; unlabeled ok)	Medium	Faster transfer across products; anomalies missed by single‐process models (often qualitative)
Integrated multimodal (vision plus sensors plus text; concept)	End‐to‐end safety assistant	Video plus sensor logs plus incidents (TB scale)	Very high	Conceptual; early prototypes suggest cross‐signal flags

Abbreviations: CNN, convolutional neural network; HACCP, hazard analysis and critical control points; LLM, large language model; ViT, vision transformer.

## 6 Real‐Time Systems, Validation, and Regulation

AI models for food safety and quality must ultimately run in production environments—often at the *edge* (on factory floors, in fields, or in distribution centers)—and comply with rigorous validation standards and regulations. This section covers hardware acceleration for edge deployment, techniques to compress and optimize models, system engineering (MLOps) in operational settings, validation and benchmarking of model performance, documentation and auditability, and the regulatory/standards landscape. The emphasis is on peer‐reviewed evidence of deploying AI *outside* the laboratory, ensuring reliability and compliance in real‐world food systems.

### 6.1 Hardware and Acceleration at the Edge

#### 6.1.1 Edge Computing Requirements

Food manufacturing and supply chain environments impose constraints on AI hardware. Devices may need to be compact, power‐efficient, and rugged (with water‐proof or wash‐down enclosures meeting IP65/IP69K for hygiene) to survive in wet, cold, or clean‐in‐place settings. For instance, smart cameras on processing lines are often enclosed in stainless steel housings to allow daily sanitization (Digital Twin in Food Manufacturing: Gamechanger or Hype?, n.d.). Embedded GPUs (like NVIDIA Jetson series), NPUs (neural processing units), and FPGAs are commonly evaluated for running vision models on the line. These accelerators can perform inference with low latency (< 100 ms), (Liu et al. 2025) which is critical for real‐time rejection of contaminated products. A case study in a dairy plant used an NVIDIA Jetson Xavier to run a CNN that checks bottle fill levels at 30 bottles/s; the device's small form factor allowed it to be mounted directly on the conveyor, avoiding network latency, and its GPU could process each image in 10 ms.

#### 6.1.2 Throughput and Latency Benchmarks

Heffer et al. reported deploying their multi‐sensor spoilage model on an edge PC; it achieved inference in under 1 s per sample, meeting the real‐time requirement for a conveyor moving at 1 sample/s (Heffer et al. 2025). Similarly, Liu et al. achieved *25.7 FPS* on a Jetson Xavier for a multimodal pest detection model by using an optimized lightweight network (Liu et al. 2025). Edge FPGAs have been used for even faster throughput when dealing with simple models: Enériz et al. implemented an e‐nose signal classifier on a low‐cost FPGA, which could classify milk quality in *2 ms* after each sensor reading—effectively real‐time for monitoring spoilage progression (Enériz et al. 2021).

#### 6.1.3 Thermal and Environmental Considerations

Edge devices in food plants may face temperature extremes (e.g., freezers or ovens) and must dissipate heat. Passive cooling is preferred to avoid fans (which can harbor contaminants). Researchers have tested devices like Google Coral TPU and Jetson Nano in cold rooms (4°C) and found performance stable, but noted that in hot environments (near ovens) TPU throttling occurred above 50°C ambient, necessitating heat sinks or relocation away from heat sources (Monteiro and Barata 2025). FPGAs tend to have lower power draw and thus less heat, which can be an advantage in sealed enclosures with no active cooling.

#### 6.1.4 I/O With Sensors

Integration of cameras (visible, thermal, HSI), e‐noses, and other sensors at the edge requires adequate I/O bandwidth. High‐speed USB3 or GigE for cameras and serial/analog interfaces for chemical sensors are typical. There is evidence that moving processing closer to the sensor (on the device) reduces data bottlenecks—for example, performing partial image analysis on‐camera before sending results downstream. In one approach, a smart hyperspectral camera did on‐board feature extraction (converting a 100‐band cube to 3 key feature images), then transmitted those to the edge computer, cutting data size by 95% and enabling real‐time inspection at 60 Hz (Jiang et al. 2025).

#### 6.1.5 Ruggedization

Few peer‐reviewed papers discuss this in detail, but industrial reports indicate AI devices must be IP‐rated (dust/water proof) and sometimes explosion‐proof (for grain dust environments). Some studies note using conformal coating on circuit boards to protect against moisture and food acids, and choosing food‐grade materials for enclosures. Wires and connectors also need protection from frequent plugging/unplugging during washdowns. These practical aspects often determine whether an AI solution can actually be deployed long‐term on a factory floor.

### 6.2 Deploying Large Models: Quantization, Pruning, and Streaming

Large neural models, while powerful, can be impractical to deploy on the edge without compression (Table 5).

**TABLE 5:** Edge deployment trade‐offs and design choices, with reported performance impacts.

Constraint	Mitigation	Reported impact (example)
Low latency (under 100 ms)	Compact or early‐exit models; INT8 quantization; optimized inference (e.g., TensorRT)	30 ms vs. 100 ms with less than 1% accuracy loss; early‐exit 60 ms for most cases, 90 ms worst‐case
Limited compute (ARM, small GPU)	Pruning; distillation; cloud offload (if allowed)	Pruned model kept F1 with smaller size; distilled edge model 5% lower accuracy but 5× faster; cloud added 200 ms latency
Harsh environment (washdown, heat, humidity)	IP‐rated enclosures; passive cooling; low‐thermal hardware; redundancy	Reliability gains; thermal throttling increased inference time by 20% until cooling improved
Limited bandwidth (remote sites)	On‐device features only; compression; buffer and sync	95% data reduction with no detection loss (feature transmit); batch upload enabled daily reports; sorting improved 15%
Power constraints (battery, solar)	Low‐power hardware; sparsification; duty cycling	FPGA under 5 W; duty cycle cut power 50% with 90% event capture

#### 6.2.1 Quantization

Reducing model weight precision from 32‐bit floats to 16‐bit or 8‐bit integers can dramatically shrink model size and speed up inference on specialized hardware (TPUs, NPUs) with minimal accuracy loss. In a food image classification example, quantizing a ResNet50 to 8‐bit resulted in only a 0.5% drop in accuracy for fruit defect detection, while doubling inference speed on an ARM CPU (Guo et al. 2024; Liu et al. 2025). Low‐precision inference is especially effective on vision models; however, for some spectral regression tasks, quantization can hurt performance if the model needs fine numeric precision. Researchers found that an 8‐bit quantized model predicting wine metabolite levels had 2% higher error than a 32‐bit model, likely due to cumulative rounding affecting the precise regression output (Guo et al. 2024). Mixed precision (keeping critical layers in higher precision) is one solution.

#### 6.2.2 Pruning

Pruning removes redundant weights or filters in the network. A study on a meat freshness CNN pruned 30% of convolutional filters (based on lowest activation contributions) and saw almost no change in classification accuracy (within 1%) (Qiu et al. 2025). This reduced model size allows deployment on a microcontroller. However, aggressive pruning (50%+) began to drop accuracy noticeably (3%–5%), showing a tradeoff. Structured pruning (removing entire channels) tends to be hardware‐friendly and easier to optimize on GPUs. In one case, pruning led to a model small enough to fit on an FPGA, enabling inference at the point of sensing (an “AI‐on‐sensor” approach for an e‐nose) (Enériz et al. 2021).

#### 6.2.3 Distillation

Large models can be distilled into smaller models by training a compact “student” model to replicate the outputs of the large “teacher” model. This was done for a food image segmentation transformer: A ResNet‐based U‐Net student was trained on the outputs of a big vision transformer on a food contaminant segmentation task, achieving 95% of the large model's IoU score while being 5× faster and able to run on a mobile GPU (Sui et al. 2026). Distillation works well when a large model exists but is too slow for real‐time needs. It also implicitly transfers some robustness of the teacher to the student.

#### 6.2.4 Operator Fusion and Optimized Runtimes

On the software side, deploying models through optimized inference engines (TensorRT, OpenVINO, etc.) can yield big speed‐ups. These tools perform operator fusion (combining sequences of operations into one for efficiency) and target‐specific optimizations. In a packaging inspection AI, using TensorRT on a Jetson yielded a 3× speed boost vs. using TensorFlow out‐of‐the‐box, allowing the system to meet the 50 ms per image requirement (20 FPS). Many peer works mention using such toolkits, although details are often relegated to implementation notes.

#### 6.2.5 Streaming Inference

When dealing with video or continuous sensor streams, sliding window or streaming architectures avoid re‐computation. For example, rather than running a fresh inference on each video frame independently, one can use a rolling state. An RNN or 1D CNN can be applied in a streaming fashion for sensor data: a study on real‐time fermentation monitoring used a streaming LSTM that updated its prediction of fermentation stage with each new sensor reading rather than reprocessing the entire time‐series each time; this reduced latency and allowed detection of deviations within seconds of them occurring (Ponzo et al. 2024). Similarly, pipeline parallelism can be employed: while one frame is being analyzed by later layers of a CNN, the next frame is entering the early layers—maximizing throughput on edge accelerators. The impact on accuracy of these deployment tricks is usually negligible in steady‐state, though one must ensure that stateful streaming models don't accumulate error over long runs (periodic resets or drift correction may be needed). In summary, the literature demonstrates multiple viable strategies to compress and deploy models: quantization and pruning for size/latency, distillation to leverage large‐model performance in small models, and efficient inference pipelines—all crucial for bringing AI from the lab to the field in food systems.

### 6.3 Systems Engineering and MLOps in Factories

Beyond the model itself, deploying AI in production requires a whole system to manage data, models, and feedback loops—often referred to as MLOps (Machine Learning Operations).

#### 6.3.1 Deterministic Pipelines and Synchronization

Food factories typically prefer deterministic, validated processes. AI pipelines must integrate with existing line equipment and PLCs (programmable logic controllers). For example, if a vision system is used on a sorting line, it might trigger a reject actuator. Ensuring synchronization (that the right object is rejected) is critical. Researchers implementing a real‐time apple sorting AI described a deterministic scheduling where each image frame was stamped with an item ID and the inference result was passed to the PLC controlling the rejection arm with a fixed offset in time. Buffering was used so that even if occasional frames were dropped, each apple's decision was held until the exact moment it reached the rejection point. This deterministic design satisfied the plant engineers' requirements for traceability of each item's outcome.

#### 6.3.2 Versioning and CI/CD for Models

When models are updated (re‐trained on new data or improved), rigorous version control is needed. One case study from a beverage company reported using a model registry to keep track of model versions deployed on different production lines. Each model was tagged with the dataset and the preprocessing version it was trained on. They employed continuous integration (CI) tests where any new model candidate had to run on a hold‐out validation set and meet performance criteria *and* speed/throughput criteria before it could be deployed. Deployment (CD) was often manual due to validation requirements, but containerization (Docker images of the inference service) helped roll back if an issue occurred. In the literature, specific examples include an AI system for bakery product inspection that underwent four version updates over a year; each update was first shadow‐deployed (running in parallel to the old model without affecting decisions) to collect comparative performance data for at least 1 month. Only after verifying the new model's outputs matched or exceeded the old one (and no new failure modes) was it switched to active use. This cautious approach mirrors practices in other industries like healthcare.

#### 6.3.3 Monitoring and Data Drift

Once deployed, models must be monitored for input data drift or performance degradation. An example is a grain sorting AI that, after a year, started showing increased false rejects because a new variety of grain with slightly different color was introduced. The system flagged this drift by monitoring the distribution of extracted features and seeing a shift outside the original training range. This triggered an alarm to data scientists, who retrained the model with samples of the new variety. Monitoring can include simple checks (e.g., average brightness of images, to detect camera illumination changes) and more complex ones (like a drop in confidence scores, which could indicate novel inputs).

#### 6.3.4 Incident Logging and Postmortems

Food safety is unforgiving of errors, so any AI mistake (e.g., a contaminated product passing through undetected) prompts investigation. AI systems should log as much as feasible: raw inputs (or features) for cases where the model's decision was close to the threshold, the decision and confidence, and system status. One paper on an AI quality control system emphasized the importance of decision logs: every decision was time‐stamped, with the model version and sensor readings saved to an audit database. When an incident occurred (e.g., a customer complaint of a defective product that passed inspection), they could retrieve the log for that item, examine what the AI saw and decided, and perform a root cause analysis (was the sensor dirty? was the model confident or did it flag uncertainty and get overridden?). Such postmortems sometimes reveal needed changes (for instance, discovering the model was not trained on a certain rare defect).

#### 6.3.5 Human‐In‐The‐Loop Fail‐Safes

Systems engineering often includes fallbacks—if the AI is uncertain or the system detects an anomaly in the AI pipeline (like too many consecutive rejections or sensor failure), a human inspection is triggered or the line is stopped. This kind of interlock is described in a case where an AI for metal detection in chocolate would pause the line if it detected metal but also if it experienced any runtime error or unusually high sensor noise, ensuring no unchecked product continued down the line in those cases. MLOps in food thus heavily emphasizes reliability, traceability, and a blending of AI with traditional automation, rather than fully autonomous operation with no oversight.

### 6.4 Method Validation and Benchmarking

Adopting AI for food safety/quality requires demonstrating that it performs at least as reliably as established methods (Table 6). In food safety, established methods (e.g., microbiological plating, chemical assays) have well‐defined validation metrics like sensitivity, specificity, limit of detection (LoD), repeatability, and reproducibility. AI‐based methods need analogous validation.

**TABLE 6:** Validation and compliance checklist.

Aspect	What to document	Example outcome (reported)
Task and risk	Decision role (CCP vs. quality); FN and FP consequences; assistive vs. autonomous; fail‐safe plan	CCP use treated as higher risk; layered controls can reduce residual risk
Performance targets	Pre‐set targets; pilot or validation results; confidence intervals; comparison vs. current method	Defect detection validated vs. humans; spoilage RMSE comparable to reference variability
Repeatability and reproducibility	Repeat runs; different days, operators, sites; calibration controls	High repeatability on repeated runs; modest drop across sites after calibration
Reference method comparison	Paired testing vs. gold standard; discrepancy review; non‐inferiority evidence	Agreement within specified tolerance; rare FN cases described and justified
Documentation and traceability	Model version and scope; data summary; decision logs; calibration and maintenance logs; change control	“Model dossier” for audits; batch logs stored; update validated and filed
Human oversight and audits	Named owner; review process; override path; routine challenge tests	Weekly spiked tests tracked; exceptions triggered retraining; alerts acknowledged by supervisor

Abbreviations: AI, artificial intelligence; CCP, critical control point; FN, false negative; FP, false positive; QA, quality assurance.

#### 6.4.1 Repeatability and Reproducibility

This refers to whether the model gives consistent results under the same conditions (repeatability) and across different conditions (reproducibility). In an interlab study, two identical vision models were deployed in two fruit packing facilities to grade apples. They were trained on the same data, but the lighting differed slightly between sites. The study reported a kappa agreement of 0.92 between the two systems' grading outputs, indicating high reproducibility, but noted that without careful color calibration, the agreement dropped to 0.85. This demonstrates that *instrument calibration* (e.g., camera color profiles) is crucial for the reproducibility of AI decisions. Some papers propose standardizing a validation protocol akin to laboratory methods—e.g., running the AI on a reference set of samples (with known “true” status by gold‐standard tests) across multiple days and operators, then checking metrics stability. For instance, a vision AI for capsule inspection was tested by three different operators capturing images, and it achieved consistent 95%–97% defect detection rates regardless of operator (showing operator‐independence, a form of reproducibility).

#### 6.4.2 LoD Analogues

In detection tasks (foreign bodies, adulterants), defining a limit of detection for AI is important. For an X‐ray+vision system detecting glass in jam, researchers defined LoD as the smallest glass piece that could be detected with ≥ 95% probability. They found LoD = 2 mm for their AI system, which they benchmarked against the LoD of human inspectors (3–4 mm under factory conditions). Similarly, for chemical hazards predicted by spectral AI, LoD might be the lowest concentration of adulterant where the model's recall is at least 90%. One milk adulteration model had an LoD of 0.5% water added (meaning below that it frequently missed), which was deemed acceptable since regulatory tolerance was 1%. These quantitative thresholds help decide if an AI is fit for purpose or needs improvement.

#### 6.4.3 ROC/PR Curves and Confusion Costs

Many papers report AUROC (Area Under ROC Curve) or AUPRC for their models on test sets. While useful, in food safety often a high sensitivity (few false negatives) is paramount, even at the cost of more false positives. Thus, setting the operating point involves choosing a threshold that meets a target sensitivity. Validation might involve ensuring sensitivity ≥ 99% (if that's required to catch nearly all hazards) and then measuring the resulting false‐positive rate. A peer‐reviewed evaluation of an AI allergen detector (which predicts if a product contains an undeclared allergen from its image and ingredients list) showed that by tuning the threshold, they achieved 99% sensitivity but had 5% false alerts. They deemed this acceptable as a screening tool (false positives lead to manual checks, which are manageable).

#### 6.4.4 Cross‐Site and Cross‐Instrument Studies

Some literature has begun to test AI models on *external validation sets*, analogous to how diagnostic devices are validated on independent cohorts. For example, a pork freshness model trained on data from one abattoir was tested on samples from another abattoir: It still ranked samples by freshness correctly in 88% of cases, versus 95% on the original domain. Any drop informs the need for either more training diversity or domain adaptation. Another study had multiple instruments: an HSI‐based fish quality model was developed on one hyperspectral camera and tested on a second camera of similar spec—it required a standardization step (adjusting for slight wavelength response differences) to maintain performance. Such multi‐instrument validation is crucial if the technology is to be scaled.

#### 6.4.5 Benchmarking Versus Existing Methods

Ultimately, peer‐reviewed analyses compare AI to current standard methods. For instance, an AI that predicts bacterial count from sensors was benchmarked against actual plate counts across 50 meat samples; the AI's RMSE was equivalent to ±0.3 log CFU, which was on par with the variability of the plating method itself. In authenticity testing, an AI that classifies via spectroscopy might be benchmarked against a targeted chemical test; if the AI can flag adulteration with 95% accuracy and the chemical test is 98% accurate, one must consider if the convenience and speed of AI justify a slight drop in accuracy. Such comparisons are starting to appear in food science journals. The consensus is that AI methods should undergo validation trials similar to lab methods—including blind testing on independent samples—before being trusted for critical control decisions. This is an ongoing area, with initiatives calling for standard validation guidelines for AI in food safety (analogous to FDA validation protocols for diagnostic devices).

### 6.5 Regulatory and Standards Landscape

As AI is incorporated into food safety and quality control, regulators are evaluating how to classify and control these systems. Importantly, peer‐reviewed analyses (rather than just policy documents) provide insight into likely regulatory approaches.

#### 6.5.1 Risk Classification of Food AI Systems

The forthcoming EU AI Act establishes a risk‐based classification for AI. Systems for food safety likely fall under “high‐risk” AI, because they affect consumer safety. A recent review by Balta et al. argues that food safety AIs (e.g., those determining product release) should indeed be considered high‐risk and thus subject to strict requirements on transparency, robustness, and human oversight. High‐risk classification will mean these AI systems must undergo conformity assessments before deployment in the EU. In the US, while there isn't an AI‐specific law yet, the FDA has started treating some food‐related AI like it does process control equipment. One article in a law‐tech journal noted that if an AI effectively controls a CCP, it might require validation akin to a *Process Authority* validation in the US—essentially proving to regulators that the process (with AI) consistently produces safe outcomes.

China's regulatory landscape for food safety is anchored in a national legal framework and implemented through a standards‐driven compliance system (Food Safety Law of the PRC (2015), n.d.). The Food Safety Law establishes the national approach to risk monitoring, risk assessment, and the role of national food safety standards in underpinning supervision and administration (Food Safety Law of the People's Republic of China, n.d.; Food Safety Law of the PRC (2015), n.d.). In practice, compliance requirements are operationalized through National Food Safety Standards (GB), with major updates jointly issued by the National Health Commission (NHC) and the State Administration for Market Regulation (SAMR), including revisions to prepackaged food labelling standards and nutrition labelling requirements (e.g., GB 7718‐2025; GB 28050‐2025) and contaminant limits (e.g., GB 2762‐2025) (National Health Commission of the People's Republic of China, and Department of Food Safety Standards and Monitoring Evaluation 2025; China: Nutritional Labeling Standards for Prepackaged Food Finalized|USDA Foreign Agricultural Service, n.d.). National‐level risk assessment capacity is supported by the China National Center for Food Safety Risk Assessment (CFSA) under the National Health Commission, which provides technical support for food safety risk assessment and standard‐setting (Wu et al. 2018; Yang et al. 2023). For AI‐enabled monitoring systems, this implies that validation plans and performance claims should be mapped to relevant GB requirements (where applicable), supported by auditable documentation suitable for inspection and enforcement, and designed to remain robust under practical sources of variability (site, season, instrument, and drift).

#### 6.5.2 Data Governance and Privacy

Food companies also handle a lot of data (images, sensor data, perhaps worker activity on lines). The EU General Data Protection Regulation (GDPR) and similar laws might come into play, for instance, if cameras inadvertently record employees. One peer‐reviewed commentary raised the point that AI systems should be assessed for privacy impact; if employee actions are being monitored (even indirectly, like seeing if a manual cleaning step was done properly via computer vision), workers might need to be informed and their consent or union agreement obtained. However, most food quality AIs focus on products, not people, so privacy is usually less of a concern than in consumer‐facing AI.

#### 6.5.3 Standards and Guidelines

There are efforts to develop technical standards for AI in food. For example, ISO and IEEE are working on guidelines for AI in manufacturing, which would cover performance metrics, testing, and algorithm transparency. A 2025 analysis in a food control journal highlighted the alignment needed between AI validation and ISO 22000 food safety management systems. It suggested that companies integrate AI performance monitoring into their ISO documentation so that it is part of the certified quality system. Another aspect is standardizing the datasets for evaluation—for example, an idea floated is a public repository of known‐good and known‐defect product images that any new inspection AI should be tested against, akin to standard test strains for pathogen detection methods.

#### 6.5.4 Postmarket Monitoring

Regulatory science literature emphasizes that AI models may change or degrade over time, so regulators might require continued monitoring and periodic re‐validation. The FDA's perspective (from a 2025 workshop on regulatory science and AI) is that for adaptive or continuously learning systems, companies need to implement a change management protocol and possibly seek approval for significant model changes. In food, models are usually static after deployment (they don't update themselves without a deliberate retraining), which regulators may find easier to handle—essentially treating the model as a fixed “instrument” that should be re‐validated if changed.

#### 6.5.5 Liability and Legal Implications

Who is responsible if the AI misses a hazard and a consumer is harmed? Legal scholars (as cited by Ma et al. 2024) indicate that ultimately the food business operator remains responsible under food law. AI is viewed as a tool, and its use does not absolve companies from the duty of care. However, if AI becomes common, we might see guidelines like “if using AI for quality control, it must meet at least the sensitivity of existing methods” codified. Some jurisdictions are exploring requiring notifications if AI is used in the food safety process—so far, no such requirement exists, but regulators have asked in audits whether AI is being used and how it is validated. The consensus in literature is that regulators are cautiously supportive of AI to enhance food safety, but they demand transparency (clear documentation, perhaps algorithmic explainability for critical decisions) and risk mitigation (human oversight, fail‐safes). For instance, an EFSA (European Food Safety Authority) journal article posited that an AI decision‐making tool affecting food could be required to have a “human‐in‐the‐loop” until proven extremely reliable. In conclusion, while no specific “AI in food” regulations exist yet, applying general AI principles and existing food safety law indicates that high standards of validation, documentation, and oversight will be enforced. Companies deploying these systems should prepare for detailed scrutiny—essentially having to prove that the AI is as safe, if not safer, than the traditional methods it replaces.

#### 6.5.6 Evidence Gaps

While multimodal AI shows clear promise in food safety and quality, there are still gaps in the published evidence. Few studies report multisite industrial deployments over long periods—thus, evidence on how models handle seasonal/raw material shifts or equipment drift over years is sparse. Also, drift management strategies (like periodic recalibration or incremental learning in production) are not well documented in literature yet. Public, peer‐reviewed cost–benefit analyses are limited: industry likely evaluates ROI, but academic papers rarely quantify the economic benefit of AI vs. traditional methods (e.g., reduction in false rejects or labor saved). Additionally, open benchmark datasets for food AI are lacking—which hampers consistent evaluation of competing methods. As the field matures, addressing these gaps with shared datasets, long‐term studies, and economic analyses will be important to fully validate AI's impact in real‐world food systems.

## 7 Challenges and Future Perspectives

### 7.1 Multimodal Sensing and Data Fusion

AI‐driven food monitoring relies on combining inputs from multiple sensors (e.g., optical cameras, spectrometers, electronic noses and tongues) to capture the complex indicators of food quality and safety. Integrating these heterogeneous data streams is challenging: current systems often face data heterogeneity and lack standardized, representative datasets across different foods and regions (Balakrishnan et al. 2025; Dakhia et al. 2025). Single‐sensor approaches cannot fully capture attributes like appearance, odor, and chemistry simultaneously, so multimodal data fusion is needed for robust detection (Balakrishnan et al. 2025). However, effective fusion requires advanced algorithms and calibration across modalities. Sensor drift over time further complicates reliability, as gradual changes in sensor sensitivity can degrade model accuracy (Arun et al. 2025). These technical hurdles must be overcome to ensure that AI models can reliably integrate diverse signals into a holistic assessment of food quality.

### 7.2 Edge Deployment and Real‐Time Decision‐Making

Monitoring food safety across the supply chain often demands real‐time analysis at or near the point of sensing; for example, in farms, processing plants, or retail storage. Edge computing enables on‐site AI inference with low latency, reducing reliance on cloud connectivity (Arun et al. 2025; Gowrishankar et al. 2023; Yang et al. 2020). This is critical for time‐sensitive decisions like detecting spoilage or contamination early. Yet deploying AI on edge devices introduces constraints: Limited computing power and energy on sensors or IoT nodes necessitate lightweight models (e.g., TinyML on smart packaging) and efficient algorithms. There is an inherent trade‐off between speed and accuracy; current real‐time systems may sacrifice some accuracy to meet latency requirements (Coluccia et al. 2021; Dakhia et al. 2025; Iftikhar et al. 2022; Khan et al. 2024). Ensuring consistent performance under resource constraints and environmental extremes (temperature, humidity) remains difficult. Moreover, at‐scale deployment across many facilities raises infrastructural issues: Not all sites have reliable power and internet connectivity, particularly in developing regions, hindering uniform adoption (Balakrishnan et al. 2025). Robust edge AI architectures and fallback mechanisms are needed to guarantee uninterrupted monitoring and timely alerts in diverse conditions.

### 7.3 Model Validation, Scalability, and Regulatory Barriers

AI models for food quality must be rigorously validated to earn trust in real‐world operations. A major challenge is generalizability: models trained on limited or localized data may fail when applied to different products or regions (Balakrishnan et al. 2025). For instance, an algorithm calibrated on milk samples from one country may misclassify samples from another region with different additives or processing methods (Balakrishnan et al. 2025). Ensuring scalability across the global supply chain requires curating large, diverse training datasets, yet food safety data are often fragmented and siloed due to privacy and proprietary concerns (Balakrishnan et al. 2025; Dakhia et al. 2025). Additionally, many AI techniques (especially deep learning) act as “black boxes,” making their predictions hard to interpret. This lack of transparency undermines user and regulator confidence (Balakrishnan et al. 2025). Food industry regulators have not yet established clear guidelines for approving AI‐driven monitoring systems, creating uncertainty in compliance. Models must not only be accurate but also explainable and robust to sensor drift or environmental changes over time. Developing validation protocols (including periodic re‐calibration with reference tests) and international standards for AI in food safety will be key to overcoming these technical and regulatory barriers (Dakhia et al. 2025).

### 7.4 Future Perspectives

Despite current limitations, emerging approaches promise to enhance AI‐driven food safety monitoring. Zero‐shot learning is a frontier for improving generality: New models aim to recognize novel hazards or products without needing extensive retraining on each specific case (Guo et al. 2025). By incorporating prior knowledge (e.g., food safety ontologies or knowledge graphs), zero‐shot models could identify previously unseen contaminants or quality issues, greatly boosting adaptability of AI across different foods. Federated learning is gaining attention as a privacy‐preserving solution for data scarcity (Arun et al. 2025). In a federated system, food producers and retailers can collaboratively train shared models on distributed data (e.g., from various factories or farms) without exposing sensitive or proprietary information, thus expanding the data diversity while respecting privacy. Another promising avenue is the use of digital twins, virtual replicas of food products or supply chain processes, to simulate and predict quality degradation under different scenarios (Arun et al. 2025). Digital twins combined with AI can help test interventions (like adjusting storage conditions) in silico before applying them in reality, enhancing model validation and system optimization. Finally, proactive policy and standardization efforts are needed to support these technologies. Governments and industry bodies should establish guidelines for sensor calibration, data sharing, and AI model certification in food safety. Clear regulatory frameworks and standards will encourage wider adoption by ensuring that AI‐driven monitoring systems are reliable, interoperable, and aligned with food safety requirements. By addressing technical challenges and aligning innovation with policy, AI has the potential to deliver scalable, real‐time food quality monitoring that is both safe and globally generalizable.

## 8 Conclusions

Multimodal AI now offers a credible pathway to real time, end to end assurance of food safety and quality across fresh produce, meat, dairy, and processed products. The scientific case rests on complementarity. Optical imaging detects macroscopic defects and foreign materials. Spectroscopy captures molecular composition and adulteration. Electronic noses quantify spoilage volatiles before visible change. Biosensors target pathogens, allergens, and residues with high specificity. Process and logistics telemetry provide contextual priors for risk. When fused, these modalities reduce blind spots and deliver measurable gains over single sensors for classification, detection, and regression tasks while offering redundancy that improves safety.

Translating this capability requires disciplined data work. Harmonized acquisition, accurate co‐registration to reference assays, multisite and multiseason sampling, and evaluation splits that mimic deployment conditions are prerequisites for robustness. Proactive management of shift and drift remains essential, including calibration transfer, domain adaptation, and out‐of‐distribution safeguards. These practices, coupled with uncertainty estimation and interpretable outputs, support safe use in critical control decisions.

**FIGURE 4:**
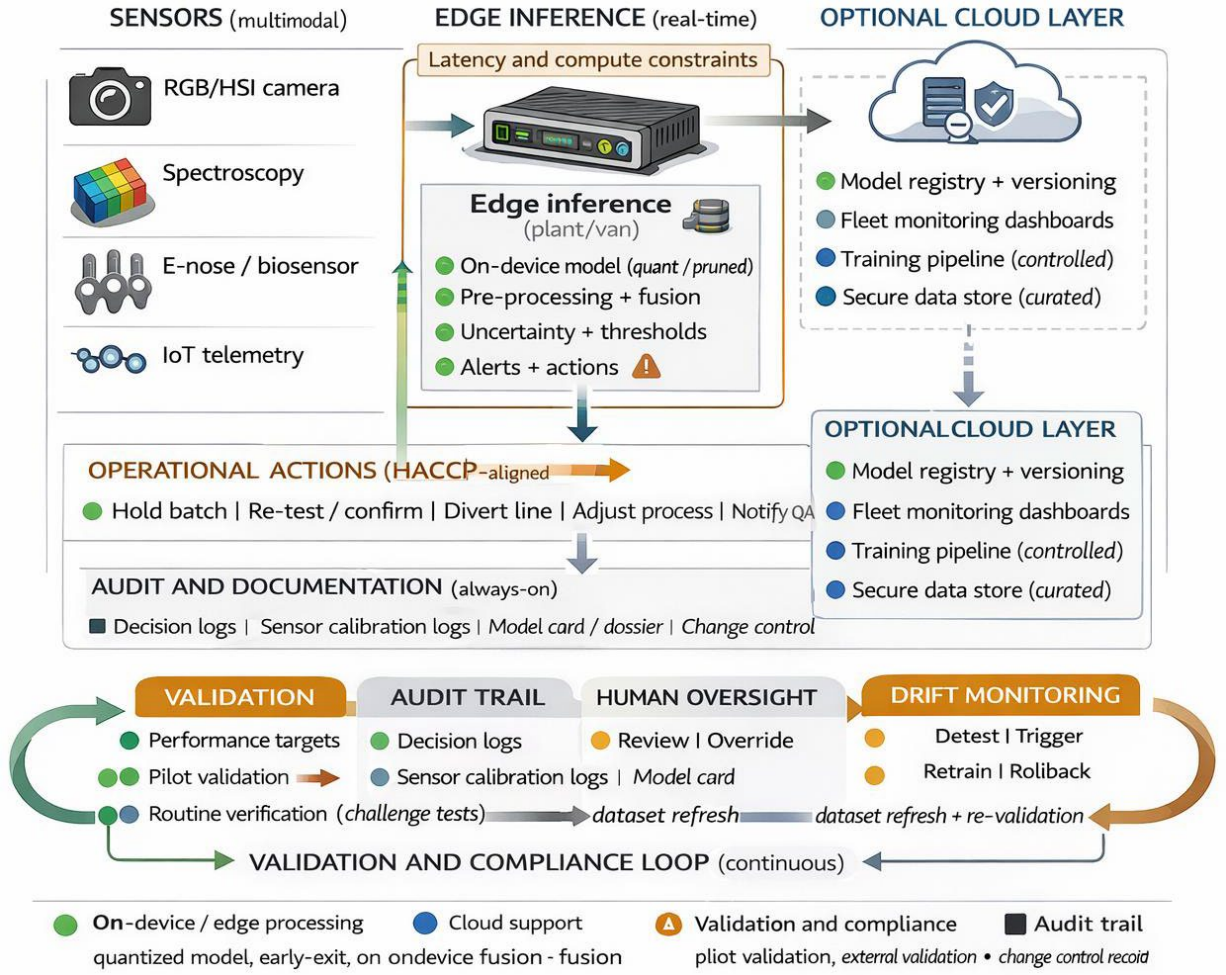
Edge deployment and compliance‐ready validation loop. Edge deployment architecture and compliance‐ready validation loop for multimodal AI in food safety and quality monitoring. Multimodal sensors feed an on‐device inference pipeline that performs pre‐processing, fusion, uncertainty‐aware decisioning, and real‐time alerting for HACCP‐aligned operational actions. Optional cloud components support model registry, controlled retraining, and fleet monitoring without compromising real‐time requirements. Auditability is maintained through persistent logging, calibration records, model documentation, and change control. Validation is treated as a lifecycle process, linking initial performance targets and external validation to routine verification and drift monitoring, with triggers for recalibration, retraining, or rollback.

Edge execution is feasible with embedded accelerators that meet throughput and hygiene constraints, provided models are optimized for latency and monitored for repeatability and reproducibility. System engineering must deliver deterministic pipelines, versioning, decision logs, and change control so that every automated release or hold can be traced to inputs, model version and rationale (Figure 4). Peer‐reviewed literature already describes practical patterns for audit readiness, from model cards and weekly challenge sets to linkage with lot records in manufacturing systems.

Looking forward, foundation scale backbones adapted with efficient tuning, knowledge infused retrieval, and active learning can accelerate coverage of diverse products and hazards, but they must be assessed for transferability, bias, and licensing constraints in regulated environments. The most pressing evidence needs are long duration, multisite industrial studies, shared multimodal benchmarks, and rigorous economic analyses of recalls averted, waste reduced, and labor reallocated. Closing these gaps will normalize auditable multimodal AI as an extension of HACCP, improving consumer protection while enhancing sustainability and competitiveness.

## Author Contributions

**Zhaojie Chen:** conceptualization, investigation, writing – original draft, methodology, validation, writing – review and editing, project administration, supervision, resources, visualization. **Guangyu Zhang:** writing, original draft, investigation, visualization. **Fan Zhang:** investigation, project administration, resources.

## Funding

The authors have nothing to report.

## Ethics Statement

The authors have nothing to report.

## Conflicts of Interest

The authors declare no conflicts of interest.

## Data Availability

The data supporting this study's findings are available from the corresponding author upon reasonable request.

## References

[fsn371534-bib-0024] 2019. “European Commission. QU4LITY –*Digital Reality in Zero Defect Manufacturing*.Horizon 2020 project; Grant agreement No. 825030. 2019–2022”. 10.3030/825030.

[fsn371534-bib-0001] Abdullahi, A. , T. Korumilli , S. Subburaj , and S. Jiga Abubakar . 2026. Spectroscopic Techniques (NIR, FTIR, and Raman) for Nutritional Profiling, 181–199. Springer US. 10.1007/978-1-0716-4787-5_9.

[fsn371534-bib-0002] Andriiashen, V. , R. van Liere , T. Leeuwen , and K. J. Batenburg . 2023. “CT‐Based Data Generation for Foreign Object Detection on a Single X‐Ray Projection.” Scientific Reports 13, no. 1: 1881. 10.1038/S41598-023-29079-W. 36732337 PMC9894971

[fsn371534-bib-0004] Aquino, A. , and C. A. Conte‐Junior . 2020. “A Systematic Review of Food Allergy: Nanobiosensor and Food Allergen Detection.” Biosensors 10, no. 12: 194. 10.3390/BIOS10120194. 33260424 PMC7760337

[fsn371534-bib-0005] Arun, A. P. , N. Sreenivasan , C. A. Rudregowda , M. Sarode , V. Moses , and R. Mudbidre . 2025. “AIoT for Smart Food Monitoring: A Review of Sensing Technologies, Edge Intelligence, and Real‐Time Quality Prediction Systems.” 2025 9th International Conference on Computational System and Information Technology for Sustainable Solutions (CSITSS), 1–5. 10.1109/CSITSS67709.2025.11294444.

[fsn371534-bib-0006] Aviara, N. A. , J. T. Liberty , O. S. Olatunbosun , H. A. Shoyombo , and S. K. Oyeniyi . 2022. “Potential Application of Hyperspectral Imaging in Food Grain Quality Inspection, Evaluation and Control During Bulk Storage.” Journal of Agriculture and Food Research 8: 100288. 10.1016/J.JAFR.2022.100288.

[fsn371534-bib-0007] Balakrishnan, P. , A. Anny Leema , N. Jothiaruna , et al. 2025. “Artificial Intelligence for Food Safety: From Predictive Models to Real‐World Safeguards.” Trends in Food Science & Technology 163: 105153. 10.1016/J.TIFS.2025.105153.

[fsn371534-bib-0008] Balta, I. , J. Lemon , C. A. Popescu , et al. 2025. “Food Safety—The Transition to Artificial Intelligence (AI) Modus Operandi.” Trends in Food Science & Technology 165: 105278. 10.1016/J.TIFS.2025.105278.

[fsn371534-bib-0009] Bannach, D. , O. Amft , and P. Lukowicz . 2009. “Automatic Event‐Based Synchronization of Multimodal Data Streams From Wearable and Ambient Sensors.” Lecture Notes in Computer Science (Including Subseries Lecture Notes in Artificial Intelligence and Lecture Notes in Bioinformatics), 5741 LNCS, 135–148. 10.1007/978-3-642-04471-7_11.

[fsn371534-bib-0010] Barazandeh, B. , S. Majumdar , O. Rajyaguru , and G. Michailidis . 2025. “*Localized LoRA: A Structured Low‐Rank Approximation for Efficient Fine‐Tuning*.” https://arxiv.org/pdf/2506.00236v2.

[fsn371534-bib-0011] Bi, J. , and C. Kuesten . 2012. “Intraclass Correlation Coefficient (ICC): A Framework for Monitoring and Assessing Performance of Trained Sensory Panels and Panelists.” Journal of Sensory Studies 27, no. 5: 352–364. 10.1111/J.1745-459X.2012.00399.X.

[fsn371534-bib-0012] Blackman, R. C. , H. C. Ho , J. C. Walser , and F. Altermatt . 2022. “Spatio‐Temporal Patterns of Multi‐Trophic Biodiversity and Food‐Web Characteristics Uncovered Across a River Catchment Using Environmental DNA.” Communications Biology 5, no. 1: 1–11. 10.1038/S42003-022-03216-z. 35322190 PMC8943070

[fsn371534-bib-0013] Bommasani, R. , D. A. Hudson , E. Adeli , et al. 2021. “*On the Opportunities and Risks of Foundation Models*.” https://arxiv.org/pdf/2108.07258.

[fsn371534-bib-0014] Bosch, S. , R. X. de Menezes , S. Pees , et al. 2022. “Electronic Nose Sensor Drift Affects Diagnostic Reliability and Accuracy of Disease‐Specific Algorithms.” Sensors 22, no. 23: 9246. 10.3390/s22239246. 36501947 PMC9740993

[fsn371534-bib-0015] Bu, Y. , J. Hu , C. Chen , et al. 2024. “ResNet Incorporating the Fusion Data of RGB & Hyperspectral Images Improves Classification Accuracy of Vegetable Soybean Freshness.” Scientific Reports 14, no. 1: 1–13. 10.1038/s41598-024-51668-6. 38297076 PMC11224382

[fsn371534-bib-0016] Chen, B. , B. Shi , J. Gong , et al. 2024. “Quality Detection and Variety Classification of Pecan Seeds Using Hyperspectral Imaging Technology Combined With Machine Learning.” Journal of Food Composition and Analysis 131: 106248. 10.1016/J.JFCA.2024.106248.

[fsn371534-bib-0017] Chen, Q. , J. Li , H. Yang , and J. Qian . 2023. “A Dynamic Shelf‐Life Prediction Method Considering Actual Uncertainty: Application to Fresh Fruits in Long‐Term Cold Storage.” Journal of Food Engineering 349: 111471. 10.1016/J.JFOODENG.2023.111471.

[fsn371534-bib-0018] Chen, T. C. , and S. Y. Yu . 2022. “The Review of Food Safety Inspection System Based on Artificial Intelligence, Image Processing, and Robotic.” Food Science and Technology Brazil 42: e35421. 10.1590/FST.35421.

[fsn371534-bib-0019] Chi, H. , C. Wang , Z. Wang , et al. 2020. “Highly Reusable Nanoporous Silver Sheet for Sensitive SERS Detection of Pesticides.” Analyst 145, no. 15: 5158–5165. 10.1039/D0AN00999G. 32725005

[fsn371534-bib-0020] n.d. “China: Nutritional Labeling Standards for Prepackaged Food Finalized|USDA Foreign Agricultural Service.” Accessed January 1, 2026. https://www.fas.usda.gov/data/china‐nutritional‐labeling‐standards‐prepackaged‐food‐finalized.

[fsn371534-bib-0021] Coluccia, B. , G. P. Agnusdei , P. P. Miglietta , and F. De Leo . 2021. “Effects of COVID‐19 on the Italian Agri‐Food Supply and Value Chains.” Food Control 123: 107839. 10.1016/J.FOODCONT.2020.107839. 33424138 PMC7775261

[fsn371534-bib-0022] Dakhia, Z. , M. Russo , and M. Merenda . 2025. “AI‐Enabled IoT for Food Computing: Challenges, Opportunities, and Future Directions.” Sensors 25, no. 7: 2147. 10.3390/S25072147. 40218659 PMC11991368

[fsn371534-bib-0023] Dias Cappelini, L. T. , O. D. Ogunbiyi , V. Guimarães Ferreira , et al. 2025. “Assessing Variability in Children's Exposure to Contaminants in Food: A Longitudinal Non‐Targeted Analysis Study in Miami, Florida.” Journal of Xenobiotics 15, no. 1: 11. 10.3390/jox15010011. 39846543 PMC11755558

[fsn371534-bib-0025] n.d. “Digital Reality in Zero Defect Manufacturing|QU4LITY|Project|Fact Sheet|H2020|CORDIS|European Commission.” Accessed October 19, 2025. https://cordis.europa.eu/project/id/825030.

[fsn371534-bib-0026] n.d. “Digital Twin in Food Manufacturing: Gamechanger or Hype?” Accessed October 19, 2025. https://www.foodnhotelasia.com/blog/fnb/digital‐twin‐in‐food‐manufacturing/.

[fsn371534-bib-0027] Ding, S. , Z. Guo , X. Chen , X. Li , and F. Ma . 2024. “DCGAN‐Based Image Data Augmentation in Rawhide Stick Products' Defect Detection.” Electronics 13, no. 11: 2047. 10.3390/ELECTRONICS13112047.

[fsn371534-bib-0028] Dyussembayev, K. , P. Sambasivam , I. Bar , J. C. Brownlie , M. J. A. Shiddiky , and R. Ford . 2021. “Biosensor Technologies for Early Detection and Quantification of Plant Pathogens.” Frontiers in Chemistry 9: 636245. 10.3389/fchem.2021.636245. 34150716 PMC8207201

[fsn371534-bib-0029] Einarsdóttir, H. , M. J. Emerson , L. H. Clemmensen , et al. 2016. “Novelty Detection of Foreign Objects in Food Using Multi‐Modal X‐Ray Imaging.” Food Control 67: 39–47. 10.1016/J.FOODCONT.2016.02.023.

[fsn371534-bib-0030] Ekramirad, N. , L. Doyle , J. Loeb , D. Santra , and A. A. Adedeji . 2024. “Hyperspectral Imaging and Machine Learning as a Nondestructive Method for Proso Millet Seed Detection and Classification.” Food 13, no. 9: 1330. 10.3390/FOODS13091330. PMC11083050 38731705

[fsn371534-bib-0031] Enériz, D. , N. Medrano , and B. Calvo . 2021. “An FPGA‐Based Machine Learning Tool for In‐Situ Food Quality Tracking Using Sensor Fusion.” Biosensors 11, no. 10: 366. 10.3390/BIOS11100366. 34677322 PMC8534206

[fsn371534-bib-0032] Femenias, A. , F. Gatius , A. J. Ramos , I. Teixido‐Orries , and S. Marín . 2022. “Hyperspectral Imaging for the Classification of Individual Cereal Kernels According to Fungal and Mycotoxins Contamination: A Review.” Food Research International 155: 111102. 10.1016/J.FOODRES.2022.111102. 35400475

[fsn371534-bib-0033] Fendor, Z. , B. H. M. van der Velden , X. Wang , A. J. Carnoli , O. Mutlu , and A. Hürriyetoğlu . 2025. “Federated Learning in Food Research.” Journal of Agriculture and Food Research 23: 102238. 10.1016/J.JAFR.2025.102238.

[fsn371534-bib-0034] Feng, H. , M. Zhang , P. Liu , Y. Liu , and X. Zhang . 2020. “Evaluation of IoT‐Enabled Monitoring and Electronic Nose Spoilage Detection for Salmon Freshness During Cold Storage.” Food 9, no. 11: 1579. 10.3390/FOODS9111579. PMC7692724 33143312

[fsn371534-bib-0035] Fodor, M. , A. Matkovits , E. L. Benes , and Z. Jókai . 2024. “The Role of Near‐Infrared Spectroscopy in Food Quality Assurance: A Review of the Past Two Decades.” Food 13, no. 21: 3501. 10.3390/FOODS13213501. PMC11544831 39517284

[fsn371534-bib-0036] Folli, G. S. , L. P. Santos , F. D. Santos , et al. 2022. “Food Analysis by Portable NIR Spectrometer.” Food Chemistry Advances 1: 100074. 10.1016/J.FOCHA.2022.100074.

[fsn371534-bib-0037] n.d. “Food Safety Law of the People's Republic of China.” Accessed January 1, 2026. https://appinchina.co/government‐documents/food‐safety‐law‐of‐the‐peoples‐republic‐of‐china/.

[fsn371534-bib-0038] n.d. “Food Safety Law of the PRC (2015).” Accessed January 1, 2026. https://www.chinalawtranslate.com/en/food‐safety‐law/.

[fsn371534-bib-0039] Friedlander, A. , and C. Zoellner . 2020. “Artificial Intelligence Opportunities to Improve Food Safety at Retail.” Food Protection Trends 40, no. 4: 272–278. https://www.foodprotection.org/publications/food‐protection‐trends/archive/2020‐07‐artificial‐intelligence‐opportunities‐to‐improve‐food‐safety‐at‐retail/.

[fsn371534-bib-0040] n.d. “Fusion of Electronic Nose and Hyperspectral Imaging for Mutton Freshness Detection Using Input‐Modified Convolution Neural Network—SUNY Empire State College.” Accessed October 18, 2025. 10.1016/j.foodchem.2022.132651. 35287109

[fsn371534-bib-0041] Gaukler, G. , M. Ketzenberg , and V. Salin . 2017. “Establishing Dynamic Expiration Dates for Perishables: An Application of RFID and Sensor Technology.” International Journal of Production Economics 193: 617–632. 10.1016/J.IJPE.2017.07.019.

[fsn371534-bib-0042] Gavai, A. , Y. Bouzembrak , W. Mu , et al. 2023. “Applying Federated Learning to Combat Food Fraud in Food Supply Chains.” NPJ Science of Food 7, no. 1: 1–9. 10.1038/s41538-023-00220-3. 37658060 PMC10474077

[fsn371534-bib-0043] Gaw, N. , S. Yousefi , and M. R. Gahrooei . 2022. “Multimodal Data Fusion for Systems Improvement: A Review.” IISE Transactions 54, no. 11: 1098–1116. 10.1080/24725854.2021.1987593.

[fsn371534-bib-0044] Giacalone, D. , I. Steen , J. Alstrup , and M. Münchow . 2020. “Inter‐Rater Reliability of “Clean Cup” Scores by Coffee Experts.” Journal of Sensory Studies 35, no. 5: e12596. 10.1111/JOSS.12596.

[fsn371534-bib-0045] Gokulkumar, K. , S. J. Huang , Y. Y. Lee , S. Kogularasu , and G. P. Chang‐Chien . 2024. “Nanoparticles of SnS on Carbon Nanofibers for Electrochemical Detection of Vanillin.” ACS Applied Nano Materials 7, no. 11: 13183–13193. 10.1021/ACSANM.4C01707.

[fsn371534-bib-0046] Göransson, M. , F. Nilsson , and Å. J. F. C. Jevinger . 2018. “Temperature Performance and Food Shelf‐Life Accuracy in Cold Food Supply Chains—Insights From Multiple Field Studies.” Food Control 86: 332–341. 10.1016/J.FOODCONT.2017.10.029.

[fsn371534-bib-0047] Gowrishankar, V. , P. Veena , P. Ponmurugan , B. Annapoorani , P. Vijayakumar , and M. Siva Ramkumar . 2023. “Edge Computing Enabled Smart Warehouse Management System for Food Processing Industries.” 14th International Conference on Computing Communication and Networking Technologies, ICCCNT 2023. 10.1109/ICCCNT56998.2023.10308398.

[fsn371534-bib-0048] Grainger, R. , T. Paniagua , X. Song , N. Cuntoor , M. W. Lee , and T. Wu . 2022. “PaCa‐ViT: Learning Patch‐To‐Cluster Attention in Vision Transformers.” Proceedings of the IEEE Computer Society Conference on Computer Vision and Pattern Recognition, 2023‐June, 18568–18578. 10.1109/CVPR52729.2023.01781.

[fsn371534-bib-0049] Gretton, A. , K. M. Borgwardt , M. J. Rasch , A. Smola , B. Schölkopf , and A. Smola GRETTON . 2012. “A Kernel Two‐Sample Test.” Journal of Machine Learning Research 13, no. 25: 723–773.

[fsn371534-bib-0050] Guo, C. , J. Zhang , W. Cai , and X. Shao . 2023. “Enhancing Transferability of Near‐Infrared Spectral Models for Soluble Solids Content Prediction Across Different Fruits.” Applied Sciences 13, no. 9: 5417. 10.3390/APP13095417.

[fsn371534-bib-0051] Guo, L. , X. Hu , W. Liu , and Y. Liu . 2025. “Zero‐Shot Detection of Visual Food Safety Hazards via Knowledge‐Enhanced Feature Synthesis.” Applied Sciences 15, no. 11: 6338. 10.3390/APP15116338.

[fsn371534-bib-0052] Guo, L. , T. Wang , Z. Wu , et al. 2020. “Portable Food‐Freshness Prediction Platform Based on Colorimetric Barcode Combinatorics and Deep Convolutional Neural Networks.” Advanced Materials 32, no. 45: 2004805. 10.1002/ADMA.202004805. 33006183

[fsn371534-bib-0053] Guo, M. , K. Wang , H. Lin , L. Wang , L. Cao , and J. Sui . 2024. “Spectral Data Fusion in Nondestructive Detection of Food Products: Strategies, Recent Applications, and Future Perspectives.” Comprehensive Reviews in Food Science and Food Safety 23, no. 1: 1–23. 10.1111/1541-4337.13301. 38284587

[fsn371534-bib-0055] Heffer, S. , M. Anastasiadi , G. J. Nychas , and F. Mohareb . 2025. “Fusion vs. Isolation: Evaluating the Performance of Multi‐Sensor Integration for Meat Spoilage Prediction.” Food 14, no. 9: 1613. 10.3390/foods14091613. PMC12071527 40361695

[fsn371534-bib-0056] Heng, Y. , Y. Zhou , D. H. Nguyen , V. D. Nguyen , and M. Jiao . 2025. “An Electronic Nose Drift Compensation Algorithm Based on Semi‐Supervised Adversarial Domain Adaptive Convolutional Neural Network.” Sensors and Actuators B: Chemical 422: 136642. 10.1016/J.SNB.2024.136642.

[fsn371534-bib-0057] Heo, J. , and S. Z. Hua . 2009. “An Overview of Recent Strategies in Pathogen Sensing.” Sensors 9, no. 6: 4483. 10.3390/S90604483. 22408537 PMC3291922

[fsn371534-bib-0058] Heusel, M. , H. Ramsauer , T. Unterthiner , B. Nessler , and S. Hochreiter . 2017. “GANs Trained by a Two Time‐Scale Update Rule Converge to a Local Nash Equilibrium.” Advances in Neural Information Processing Systems 30: 6627–6638. 10.48550/arXiv.1706.08500.

[fsn371534-bib-0059] Hyland, S. L. , E. Zurich , C. Esteban , and G. Rätsch . 2017. “Real‐valued (Medical) Time Series Generation with Recurrent Conditional GANs.” https://arxiv.org/pdf/1706.02633.

[fsn371534-bib-0060] n.d. “Hy‐Vee Grocery Cold Chain Monitoring Increases Product Shelf‐Life.” Accessed October 17, 2025. https://www.impinj.com/library/customer‐stories/hy‐vee‐grocery‐cold‐chain‐monitoring‐increase.

[fsn371534-bib-0061] Iftikhar, A. , I. Ali , A. Arslan , and S. Tarba . 2022. “Digital Innovation, Data Analytics, and Supply Chain Resiliency: A Bibliometric‐Based Systematic Literature Review.” Annals of Operations Research 333, no. 2: 825–848. 10.1007/S10479-022-04765-6. PMC9118819 35611176

[fsn371534-bib-0062] n.d. “ISO 13299:2016—Sensory Analysis—Methodology—General Guidance for Establishing a Sensory Profile.” Accessed December 31, 2025. https://www.iso.org/standard/58042.html.

[fsn371534-bib-0063] n.d. “ISO 8589:2007—Sensory Analysis—General Guidance for the Design of Test Rooms.” Accessed December 31, 2025. https://www.iso.org/standard/36385.html.

[fsn371534-bib-0064] Jiang, W. , C. Liu , W. Liu , and L. Zheng . 2025a. “Advancements in Intelligent Sensing Technologies for Food Safety Detection.” Research (Washington, D.C.) 8: 713. 10.34133/RESEARCH.0713. PMC12128931 40458611

[fsn371534-bib-0066] Jin, S. , X. Liu , J. Wang , et al. 2023. “Hyperspectral Imaging Combined With Fluorescence for the Prediction of Microbial Growth in Chicken Breasts Under Different Packaging Conditions.” LWT 181: 114727. 10.1016/J.LWT.2023.114727.

[fsn371534-bib-0067] Jixiang, E. , C. Zhai , X. Jiang , Z. Xu , M. Wudan , and D. Li . 2025. “Non‐Destructive Detection of Chilled Mutton Freshness Using a Dual‐Branch Hierarchical Spectral Feature‐Aware Network.” Food 14, no. 8: 1379. 10.3390/FOODS14081379. PMC12027296 40282781

[fsn371534-bib-0068] Kabir, M. H. , Z. Zhang , X. Li , et al. 2025. “Full‐Surface Detection of Apple Fruits Using Enhanced YOLOv5.” Agricultural Products Processing and Storage 1, no. 1: 1–19. 10.1007/S44462-025-00020-W.

[fsn371534-bib-0069] Khan, N. , W. D. Solvang , and H. Yu . 2024. “Industrial Internet of Things (IIoT) and Other Industry 4.0 Technologies in Spare Parts Warehousing in the Oil and Gas Industry: A Systematic Literature Review.” Logistics 8, no. 1: 16. 10.3390/LOGISTICS8010016.

[fsn371534-bib-0070] Kim, K. , H. Kim , J. Chun , M. Kang , M. Hong , and B. Min . 2021. “Real‐Time Anomaly Detection in Packaged Food X‐Ray Images Using Supervised Learning.” Computers, Materials & Continua 67, no. 2: 2547–2568. 10.32604/CMC.2021.014642.

[fsn371534-bib-0071] Kramer, K. E. 2005. “*Improving the Robustness of Multivariate Calibration Models for the Determination of Glucose by Near‐Infrared Spectroscopy*.” 10.17077/ETD.FAY4YG6J.

[fsn371534-bib-0072] Kuesten, C. , and J. Bi . 2021. “Application of a Panel Performance Reliability Versus Product Effect Size (PR‐ES) Framework: A Protein Powder Case Study.” Food Quality and Preference 89: 104152. 10.1016/J.FOODQUAL.2020.104152.

[fsn371534-bib-0073] Kuhl, E. 2025. “AI for Food: Accelerating and Democratizing Discovery and Innovation.” NPJ Science of Food 9, no. 1: 1–10. 10.1038/s41538-025-00441-8. 40404647 PMC12098880

[fsn371534-bib-0074] Kurniawan, H. , M. A. A. Arief , S. Lohumi , M. S. Kim , I. Baek , and B. K. Cho . 2024. “Dual Imaging Technique for a Real‐Time Inspection System of Foreign Object Detection in Fresh‐Cut Vegetables.” Current Research in Food Science 9: 100802. 10.1016/J.CRFS.2024.100802. 39100806 PMC11294706

[fsn371534-bib-0075] Kynkäänniemi, T. , T. Karras , S. Laine , J. Lehtinen , and T. Aila . 2019. “Improved Precision and Recall Metric for Assessing Generative Models.” Advances in Neural Information Processing Systems 32: 3927–3936. https://arxiv.org/pdf/1904.06991.

[fsn371534-bib-0076] Lei, J. , K. He , X. Sun , Z. Yuan , and J. Zhang . 2025. “Adaptive Multimodal Fusion via Attention‐Guided Feature Selection for Histopathology Image Classification.” 163–168. 10.1109/ICIP55913.2025.11084638.

[fsn371534-bib-0077] Li, C. , M. He , Z. Cai , H. Qi , J. Zhang , and C. Zhang . 2023. “Hyperspectral Imaging With Machine Learning Approaches for Assessing Soluble Solids Content of Tribute Citru.” Food 12, no. 2: 247. 10.3390/FOODS12020247. PMC9857513 36673336

[fsn371534-bib-0078] Li, P. , X. Huang , Y. Tian , and N. V. Chawla . 2024. “ChefFusion: Multimodal Foundation Model Integrating Recipe and Food Image Generation.” Proceedings of the 33rd ACM International Conference on Information and Knowledge Management (CIKM '24), October 21â•fi25, 2024, Boise, ID, USA, 1. 10.1145/3627673.3679885.

[fsn371534-bib-0079] Lim, H. , and E. Song . 2025. “Beef Carcass Grading With EfficientViT: A Lightweight Vision Transformer Approach.” Applied Sciences 15, no. 11: 6302. 10.3390/APP15116302.

[fsn371534-bib-0080] Lim, J. , A. Wood , and B. G. Green . 2009. “Derivation and Evaluation of a Labeled Hedonic Scale.” Chemical Senses 34, no. 9: 739. 10.1093/CHEMSE/BJP054. 19833660 PMC2762053

[fsn371534-bib-0081] Lin, Y. , J. Ma , D. W. Sun , J. H. Cheng , and C. Zhou . 2024. “Fast Real‐Time Monitoring of Meat Freshness Based on Fluorescent Sensing Array and Deep Learning: From Development to Deployment.” Food Chemistry 448: 139078. 10.1016/J.FOODCHEM.2024.139078. 38527403

[fsn371534-bib-0082] Liu, C. , Z. Chu , S. Weng , et al. 2022. “Fusion of Electronic Nose and Hyperspectral Imaging for Mutton Freshness Detection Using Input‐Modified Convolution Neural Network.” Food Chemistry 385: 132651. 10.1016/J.FOODCHEM.2022.132651. 35287109

[fsn371534-bib-0083] Liu, T. , D. Li , and J. Chen . 2021. “An Active Method of Online Drift‐Calibration‐Sample Formation for an Electronic Nose.” Measurement 171: 108748. 10.1016/J.MEASUREMENT.2020.108748.

[fsn371534-bib-0084] Liu, T. , D. Li , J. Chen , Y. Chen , T. Yang , and J. Cao . 2018. “Gas‐Sensor Drift Counteraction With Adaptive Active Learning for an Electronic Nose.” Sensors 18, no. 11: 4028. 10.3390/S18114028. 30463202 PMC6263697

[fsn371534-bib-0085] Liu, Z. , S. Li , Y. Yang , et al. 2025. “High‐Precision Pest Management Based on Multimodal Fusion and Attention‐Guided Lightweight Networks.” Insects 16, no. 8: 850. 10.3390/INSECTS16080850. 40870651 PMC12386247

[fsn371534-bib-0086] Long, Y. , W. Huang , Q. Wang , S. Fan , and X. Tian . 2022. “Integration of Textural and Spectral Features of Raman Hyperspectral Imaging for Quantitative Determination of a Single Maize Kernel Mildew Coupled With Chemometrics.” Food Chemistry 372: 131246. 10.1016/J.FOODCHEM.2021.131246. 34818727

[fsn371534-bib-0087] Lopez‐Paz, D. , and M. Oquab . 2016. “Revisiting Classifier Two‐Sample Tests.” International Conference on Learning Representations.

[fsn371534-bib-0088] Lun, Z. , X. Wu , J. Dong , and B. Wu . 2025. “Deep Learning‐Enhanced Spectroscopic Technologies for Food Quality Assessment: Convergence and Emerging Frontiers.” Food 14, no. 13: 2350. 10.3390/FOODS14132350. PMC12248972 40647102

[fsn371534-bib-0089] Lundberg, S. , and S.‐I. Lee . 2017. “A Unified Approach to Interpreting Model Predictions.” *ArXiv*. 10.48550/arXiv.1705.07874.

[fsn371534-bib-0090] Ma, L. , X. Yang , S. Xue , et al. 2025. “‘Raman Plus X’ Dual‐Modal Spectroscopy Technology for Food Analysis: A Review.” Comprehensive Reviews in Food Science and Food Safety 24, no. 1: e70102. 10.1111/1541-4337.70102. 39746858

[fsn371534-bib-0091] Ma, P. , S. Tsai , Y. He , et al. 2024. “Large Language Models in Food Science: Innovations, Applications, and Future.” Trends in Food Science & Technology 148: 104488. 10.1016/J.TIFS.2024.104488.

[fsn371534-bib-0092] Madhubhashini, M. N. , C. P. Liyanage , A. U. Alahakoon , and R. P. Liyanage . 2024. “Current Applications and Future Trends of Artificial Senses in Fish Freshness Determination: A Review.” Journal of Food Science 89, no. 1: 33–50. 10.1111/1750-3841.16865. 38051021

[fsn371534-bib-0093] Man, K. , and J. Chahl . 2022. “A Review of Synthetic Image Data and Its Use in Computer Vision.” Journal of Imaging 8, no. 11: 310. 10.3390/JIMAGING8110310. 36422059 PMC9698631

[fsn371534-bib-0094] Mehdizadeh, S. A. , M. Noshad , M. Chaharlangi , and Y. Ampatzidis . 2025. “AI‐Driven Non‐Destructive Detection of Meat Freshness Using a Multi‐Indicator Sensor Array and Smartphone Technology.” Smart Agricultural Technology 10: 100822. 10.1016/J.ATECH.2025.100822.

[fsn371534-bib-0095] Min, W. , X. Hong , Y. Liu , et al. 2025. “Multimodal Food Learning.” ACM Transactions on Multimedia Computing, Communications, and Applications 21, no. 7: 28. 10.1145/3715143.

[fsn371534-bib-0096] Min, W. , C. Liu , L. Xu , and S. Jiang . 2022. “Applications of Knowledge Graphs for Food Science and Industry.” Patterns 3, no. 5: 100484. 10.1016/J.PATTER.2022.100484. 35607620 PMC9122965

[fsn371534-bib-0097] Monteiro, J. , and J. Barata . 2025. “Digital Twin‐Enabled Regional Food Supply Chain: A Review and Research Agenda.” Journal of Industrial Information Integration 45: 100851. 10.1016/J.JII.2025.100851.

[fsn371534-bib-0098] n.d. “Multimodal Data Fusion—An Overview|ScienceDirect Topics.” Accessed October 17, 2025. https://www.sciencedirect.com/topics/computer‐science/multimodal‐data‐fusion.

[fsn371534-bib-0099] Murugesan, P. , S. Kogularasu , Y. L. Chen , Y. Y. Lee , G. P. Chang‐Chien , and M. Govindasamy . 2025. “Electrochemical Sensor for Detecting Roxarsone in Animal‐Derived Foods Using MXene and Silver Telluride.” Food Chemistry 482: 144168. 10.1016/J.FOODCHEM.2025.144168. 40187308

[fsn371534-bib-0003] National Health Commission of the People's Republic of China, and Department of Food Safety Standards and Monitoring Evaluation . 2025. “关于发布《食品安全国家标准 预包装食品标签通则》 (GB 7718‐2025) 等50项食品安全国家标准和9项修改单的公告 (2025年 第2号), March 27.” https://www.nhc.gov.cn/sps/c100088/202503/e8a432507f7d4f08a877e76a9b0578ce.shtml.

[fsn371534-bib-0100] Nicolas, L. , C. Marquilly , and M. O'Mahony . 2010. “The 9‐Point Hedonic Scale: Are Words and Numbers Compatible?” Food Quality and Preference 21, no. 8: 1008–1015. 10.1016/J.FOODQUAL.2010.05.017.

[fsn371534-bib-0101] Nikzadfar, M. , M. Rashvand , H. Zhang , et al. 2024. “Hyperspectral Imaging Aiding Artificial Intelligence: A Reliable Approach for Food Qualification and Safety.” Applied Sciences 14: 9821. 10.3390/app14219821.

[fsn371534-bib-0102] Onyeaka, H. , A. Akinsemolu , T. Miri , et al. 2025. “Artificial Intelligence in Food System: Innovative Approach to Minimizing Food Spoilage and Food Waste.” Journal of Agriculture and Food Research 21: 101895. 10.1016/J.JAFR.2025.101895.

[fsn371534-bib-0103] Pan, M. 2025. “Nondestructive Testing in Food Quality and Safety: Development and Applications.” Food 14, no. 13: 2339. 10.3390/FOODS14132339. PMC12249218 40647091

[fsn371534-bib-0104] Pan, Y. , K. Ming , D. Guo , et al. 2024. “Non‐Targeted Metabolomics and Explainable Artificial Intelligence: Effects of Processing and Color on Coniferyl Aldehyde Levels in Eucommiae Cortex.” Food Chemistry 460: 140564. 10.1016/J.FOODCHEM.2024.140564. 39089015

[fsn371534-bib-0105] Pan, Y. S. , Y. T. Chen , A. R. Nithyakrishnan , et al. 2024. “SERS‐Based Detection of Thiram in Apple Peel Samples Using Thiomalic Acid‐Ag/au Nanoparticles.” ACS Applied Nano Materials 7, no. 24: 28743–28756. 10.1021/ACSANM.4C06045.

[fsn371534-bib-0106] Ponzo, V. , R. Rosato , M. C. Scigliano , et al. 2024. “Comparison of the Accuracy, Completeness, Reproducibility, and Consistency of Different AI Chatbots in Providing Nutritional Advice: An Exploratory Study.” Journal of Clinical Medicine 13, no. 24: 7810. 10.3390/jcm13247810. 39768733 PMC11677083

[fsn371534-bib-0107] Potyrailo, R. A. , N. Nagraj , Z. Tang , F. J. Mondello , C. Surman , and W. Morris . 2012. “Battery‐Free Radio Frequency Identification (RFID) Sensors for Food Quality and Safety.” Journal of Agricultural and Food Chemistry 60, no. 35: 8535–8543. 10.1021/JF302416Y. 22881825 PMC3434321

[fsn371534-bib-0108] Pu, H. , Q. Wei , and D. W. Sun . 2023. “Recent Advances in Muscle Food Safety Evaluation: Hyperspectral Imaging Analyses and Applications.” Critical Reviews in Food Science and Nutrition 63, no. 10: 1297–1313. 10.1080/10408398.2022.2121805. 36123794

[fsn371534-bib-0109] Qiu, Z. , K. G. Liakos , V. Athanasiadis , E. Bozinou , and S. I. Lalas . 2025. “Machine Learning for Quality Control in the Food Industry: A Review.” Food 2025, no. 19: 3424. 10.3390/FOODS14193424. PMC12523314 41097592

[fsn371534-bib-0110] Quintela, I. A. , T. Vasse , C. S. Lin , and V. C. H. Wu . 2022. “Advances, Applications, and Limitations of Portable and Rapid Detection Technologies for Routinely Encountered Foodborne Pathogens.” Frontiers in Microbiology 13: 1054782. 10.3389/FMICB.2022.1054782. 36545205 PMC9760820

[fsn371534-bib-0111] Ren, R. , Z. Wang , C. Yang , et al. 2025. “Enhancing Robotic Skill Acquisition With Multimodal Sensory Data: A Novel Dataset for Kitchen Tasks.” Scientific Data 12, no. 1: 1–16. 10.1038/s41597-025-04798-z. 40118852 PMC11928623

[fsn371534-bib-0112] Röder, M. , S. Vieths , and T. Holzhauser . 2009. “Commercial Lateral Flow Devices for Rapid Detection of Peanut ( *Arachis hypogaea* ) and Hazelnut ( *Corylus avellana* ) Cross‐Contamination in the Industrial Production of Cookies.” Analytical and Bioanalytical Chemistry 395, no. 1: 103–109. 10.1007/S00216-009-2716-X. 19280180

[fsn371534-bib-0113] Rudnitskaya, A. 2018. “Calibration Update and Drift Correction for Electronic Noses and Tongues.” Frontiers in Chemistry 6: 375258. 10.3389/fchem.2018.00433. PMC6167416 30320065

[fsn371534-bib-0114] Sajjadi, M. S. M. , O. Bousquet , O. Bachem , M. Lucic , and S. Gelly . 2018. “Assessing Generative Models via Precision and Recall.” Advances in Neural Information Processing Systems 31: 5228–5237.

[fsn371534-bib-0115] Sakthivel, K. , S. Balasubramanian , G. P. Chang‐Chien , et al. 2024. “Editors' Choice—Review—Advances in Electrochemical Sensors: Improving Food Safety, Quality, and Traceability.” ECS Sensors Plus 3, no. 2: 20605. 10.1149/2754-2726/AD5455.

[fsn371534-bib-0116] Samota, S. , R. Rani , S. Chakraverty , and A. Kaushik . 2022. “Biosensors for Simplistic Detection of Pathogenic Bacteria: A Review With Special Focus on Field‐Effect Transistors.” Materials Science in Semiconductor Processing 141: 106404. 10.1016/J.MSSP.2021.106404.

[fsn371534-bib-0117] Sanislav, T. , G. D. Mois , S. Zeadally , S. Folea , T. C. Radoni , and E. A. Al‐Suhaimi . 2025. “A Comprehensive Review on Sensor‐Based Electronic Nose for Food Quality and Safety.” Sensors 25, no. 14: 4437. 10.3390/S25144437. 40732564 PMC12301011

[fsn371534-bib-0118] Sari, O. F. , E. Amer , M. Bader‐El‐Den , V. Ince , and C. Leadley . 2025. “AI‐Driven Risk Assessment in Food Safety Using EU RASFF Database.” Food and Bioprocess Technology 18, no. 7: 6282–6303. 10.1007/S11947-025-03819-4.

[fsn371534-bib-0119] Singh, R. , R. Nisha , R. Naik , K. Upendar , C. Nickhil , and S. C. Deka . 2024. “Sensor Fusion Techniques in Deep Learning for Multimodal Fruit and Vegetable Quality Assessment: A Comprehensive Review.” Journal of Food Measurement and Characterization 18, no. 9: 8088–8109. 10.1007/S11694-024-02789-Z.

[fsn371534-bib-0120] Siripatrawan, U. , and Y. Makino . 2024. “Assessment of Food Safety Risk Using Machine Learning‐Assisted Hyperspectral Imaging: Classification of Fungal Contamination Levels in Rice Grain.” Microbial Risk Analysis 27: 100295. 10.1016/J.MRAN.2024.100295.

[fsn371534-bib-0121] Soncini, N. , J. Cremona , E. Vidal , M. García , G. Castro , and T. Pire . 2025. “The Rosario Dataset v2: Multimodal Dataset for Agricultural Robotics.” International Journal of Robotics Research. 10.1177/02783649251368909.

[fsn371534-bib-0122] Stanley, M. M. , B. Sriram , S. F. Wang , V. A. Sherlin , S. Kogularasu , and M. George . 2025. “Preserving Food Quality: Electrochemical Detection of Synthetic Food Antioxidant, Propyl Gallate in Processed Foods Using Ternary Component Layered Double Hydroxide/Graphene Aerogel Synergy.” Materials Today Sustainability 29: 101061. 10.1016/J.MTSUST.2024.101061.

[fsn371534-bib-0123] Sui, W. , D. Lichau , J. Lefèvre , and H. Phelippeau . 2026. “Incomplete Multimodal Industrial Anomaly Detection via Cross‐Modal Distillation.” Information Fusion 126: 103572. 10.1016/J.INFFUS.2025.103572.

[fsn371534-bib-0124] Taitt, C. R. , Y. S. Shubin , R. Angel , and F. S. Ligler . 2004. “Detection of *Salmonella enterica* Serovar Typhimurium by Using a Rapid, Array‐Based Immunosensor.” Applied and Environmental Microbiology 70, no. 1: 152–158. 10.1128/AEM.70.1.152-158.2004. 14711637 PMC321280

[fsn371534-bib-0125] Tang, Z. , J. Lu , Z. Chen , F. Qi , and L. Zhang . 2023. “Improved Pest‐YOLO: Real‐Time Pest Detection Based on Efficient Channel Attention Mechanism and Transformer Encoder.” Ecological Informatics 78: 102340. 10.1016/J.ECOINF.2023.102340.

[fsn371534-bib-0126] Tarapoulouzi, M. , J. A. Entrenas , D. Pérez‐Marín , I. Pashalidis , and C. R. Theocharis . 2024. “A Preliminary Study on Determining Seasonal Variations in Halloumi Cheese Using Near‐Infrared Spectroscopy and Chemometrics.” PRO 12, no. 7: 1517. 10.3390/PR12071517.

[fsn371534-bib-0127] Tarlak, F. 2023. “The Use of Predictive Microbiology for the Prediction of the Shelf Life of Food Products.” Food 12, no. 24: 4461. 10.3390/FOODS12244461. PMC10743123 38137265

[fsn371534-bib-0128] n.d. “The 9‐point Hedonic Scale.” Accessed December 31, 2025. https://www.sensorysociety.org/knowledge/sspwiki/Pages/The%209‐point%20Hedonic%20Scale.aspx.

[fsn371534-bib-0129] Truong, A. M. , and H. Q. Luong . 2024. “A Non‐Destructive, Autoencoder‐Based Approach to Detecting Defects and Contamination in Reusable Food Packaging.” Current Research in Food Science 8: 100758. 10.1016/J.CRFS.2024.100758. 38779346 PMC11109354

[fsn371534-bib-0130] van Marrewijk, B. M. , S. N. Njane , S. Tsuda , et al. 2025. “TTADDA‐UAV: A Multi‐Season RGB and Multispectral UAV Dataset of Potato Fields Collected in Japan and The Netherlands.” Data in Brief 62: 112004. 10.1016/J.DIB.2025.112004. 40955419 PMC12433462

[fsn371534-bib-0131] Vikesland, P. J. , and K. R. Wigginton . 2010. “Nanomaterial Enabled Biosensors for Pathogen Monitoring—A Review.” Environmental Science & Technology 44, no. 10: 3656–3669. 10.1021/ES903704Z. 20405829

[fsn371534-bib-0132] Wang, L. , X. Sun , J. Liang , et al. 2025. “Machine Learning Model Interpretability Using SHAP Values: Applied to the Task of Classifying and Predicting the Nutritional Content of Different Cuts of Mutton.” Food Chemistry: X 29: 102739. 10.1016/J.FOCHX.2025.102739. 40686892 PMC12275142

[fsn371534-bib-0133] Wedding, B. B. , C. Wright , S. Grauf , R. D. White , B. Tilse , and P. Gadek . 2013. “Effects of Seasonal Variability on FT‐NIR Prediction of Dry Matter Content for Whole Hass Avocado Fruit.” Postharvest Biology and Technology 75: 9–16. 10.1016/J.POSTHARVBIO.2012.04.016.

[fsn371534-bib-0134] Wichchukit, S. , and M. O'Mahony . 2015. “The 9‐Point Hedonic Scale and Hedonic Ranking in Food Science: Some Reappraisals and Alternatives.” Journal of the Science of Food and Agriculture 95, no. 11: 2167–2178. 10.1002/jsfa.6993. 25378223

[fsn371534-bib-0135] Workman, J. 2025. “Universal Calibration: Can Models Travel Successfully Across Instruments?” Spectroscopy 72, no. 3: 340–365. 10.56530/SPECTROSCOPY.IE7568A1.

[fsn371534-bib-0136] Wörner, J. , J. Eimler , and M. Pein‐Hackelbusch . 2025. “Long‐Term Drift Behavior in Metal Oxide Gas Sensor Arrays: A One‐Year Dataset From an Electronic Nose.” Scientific Data 12, no. 1: 1628. 10.1038/S41597-025-05993-8. 41062497 PMC12508210

[fsn371534-bib-0137] Wu, J. , Y. Zou , Z. Chen , L. Xue , and A. Manzardo . 2025. “Reducing Potential Retail Food Waste Through a Data‐Driven Dynamic Shelf Life Approach: Insights From Consumer Engagement.” Applied Food Research 5, no. 1: 100819. 10.1016/J.AFRES.2025.100819.

[fsn371534-bib-0138] Wu, N. , J. Zhang , Q. Wang , X. Guan , and L. Zhang . 2025. “Rapid Detection of Mutton Freshness Grades and TVB‐N Using Hyperspectral Imaging With Optimized Machine Learning and Lightweight Deep Learning Models.” Journal of Food Composition and Analysis 145: 107815. 10.1016/J.JFCA.2025.107815.

[fsn371534-bib-0139] Wu, T. , H. Chen , Z. Lin , and C. Tan . 2016. “Identification and Quantitation of Melamine in Milk by Near‐Infrared Spectroscopy and Chemometrics.” Journal of Spectroscopy 2016, no. 1: 6184987. 10.1155/2016/6184987.

[fsn371534-bib-0140] Wu, Y. n. , P. Liu , and J. s. Chen . 2018. “Food Safety Risk Assessment in China: Past, Present and Future.” Food Control 90: 212–221. 10.1016/J.FOODCONT.2018.02.049.

[fsn371534-bib-0141] Wu, Z. , F. Tian , J. A. Covington , H. Li , and S. Deng . 2023. “Chemical Selection for the Calibration of General‐Purpose Electronic Noses Based on Silhouette Coefficients.” IEEE Transactions on Instrumentation and Measurement 72: 1. 10.1109/TIM.2022.3228283.37323850

[fsn371534-bib-0142] Xiao, Y. , Y. Li , G. Cui , H. Zhang , and W. Zhang . 2025. “A Systematic Review of Multimodal Fusion Technologies for Food Quality and Safety Assessment: Recent Advances and Future Trends.” Trends in Food Science & Technology 164: 105277. 10.1016/J.TIFS.2025.105277.

[fsn371534-bib-0143] Yang, D. , H. Yang , M. Shi , et al. 2023. “Advancing Food Safety Risk Assessment in China: Development of New Approach Methodologies (NAMs).” Frontiers in Toxicology 5: 1292373. 10.3389/FTOX.2023.1292373. 38046399 PMC10690935

[fsn371534-bib-0144] Yang, M. , T. Der , H. H. Tseng , Y. C. Hsu , and W. C. Tseng . 2020. “Real‐time Crop Classification Using Edge Computing and Deep Learning.” 2020 IEEE 17th Annual Consumer Communications and Networking Conference, CCNC 2020. 10.1109/CCNC46108.2020.9045498.

[fsn371534-bib-0145] Zhang, H. , Y. Lin , S. Cao , J. H. Cheng , and X. A. Zeng . 2025. “Artificial Intelligence Boosting Multi‐Dimensional Information Fusion: Data Collection, Processing and Modeling for Food Quality and Safety Assessment.” Trends in Food Science & Technology 163: 105138. 10.1016/J.TIFS.2025.105138.

[fsn371534-bib-0146] Zhang, L. , Z. Yuan , P. Li , et al. 2017. “Targeted Multivariate Adulteration Detection Based on Fatty Acid Profiles and Monte Carlo One‐Class Partial Least Squares.” Chemometrics and Intelligent Laboratory Systems 169: 94–99. 10.1016/J.CHEMOLAB.2017.09.002.

[fsn371534-bib-0147] Zhao, M. , H. Cang , H. Chen , et al. 2023. “Determination of Quality and Maturity of Processing Tomatoes Using Near‐Infrared Hyperspectral Imaging With Interpretable Machine Learning Methods.” LWT 183: 114861. 10.1016/J.LWT.2023.114861.

[fsn371534-bib-0148] Zou, Y. , J. Wu , X. Meng , X. Wang , and A. Manzardo . 2025. “Digital Twin Integration for Dynamic Quality Loss Control in Fruit Supply Chains.” Journal of Food Engineering 397: 112577. 10.1016/J.JFOODENG.2025.112577.

